# Mapping Adverse Outcome Pathways for Kidney Injury as a Basis for the Development of Mechanism-Based Animal-Sparing Approaches to Assessment of Nephrotoxicity

**DOI:** 10.3389/ftox.2022.863643

**Published:** 2022-06-15

**Authors:** Angela Mally, Sebastian Jarzina

**Affiliations:** Department of Toxicology, University of Würzburg, Würzburg, Germany

**Keywords:** adverse outcome pathway, nephrotoxicity, protein alkylation, lysosomal disruption, mitochondrial DNA polymerase γ

## Abstract

In line with recent OECD activities on the use of AOPs in developing Integrated Approaches to Testing and Assessment (IATAs), it is expected that systematic mapping of AOPs leading to systemic toxicity may provide a mechanistic framework for the development and implementation of mechanism-based *in vitro* endpoints. These may form part of an integrated testing strategy to reduce the need for repeated dose toxicity studies. Focusing on kidney and in particular the proximal tubule epithelium as a key target site of chemical-induced injury, the overall aim of this work is to contribute to building a network of AOPs leading to nephrotoxicity. Current mechanistic understanding of kidney injury initiated by 1) inhibition of mitochondrial DNA polymerase γ (mtDNA Polγ), 2) receptor mediated endocytosis and lysosomal overload, and 3) covalent protein binding, which all present fairly well established, common mechanisms by which certain chemicals or drugs may cause nephrotoxicity, is presented and systematically captured in a formal description of AOPs in line with the OECD AOP development programme and in accordance with the harmonized terminology provided by the Collaborative Adverse Outcome Pathway Wiki. The relative level of confidence in the established AOPs is assessed based on evolved Bradford-Hill weight of evidence considerations of biological plausibility, essentiality and empirical support (temporal and dose-response concordance).

## 1 Introduction

There is general consensus that comprehensive understanding of how chemicals and drugs cause adverse effects is key to the development of human relevant, animal-sparing solutions for safety testing. Adverse Outcome Pathways (AOPs) represent a formal description of the mechanistic linkage between a molecular initiating event, a series of intermediate steps and key events at different levels of biological organization, and an adverse outcome. While the concept of mode-of-action (or AOPs) has been a key aspect of human cancer risk assessment for some time, it was recently adopted by the Organisation for Economic Co-operation and Development (OECD) as a pragmatic tool which may facilitate transition of chemical safety assessment from measurement of apical endpoints in animals to toxicity prediction based on mechanistic information (Vinken, 2013). Identification of key events and systematic mapping of AOPs for a given hazard endpoint can form the basis for the development of alternative tests (*in vitro*, lower organisms, refined *in vivo*) as part of a science-based integrated testing strategy to eventually replace conventional guideline studies (Tollefsen et al., 2014; Sakuratani et al., 2018). To promote implementation of this concept into chemical safety assessment, the OECD has published guidance documents for the development, assessment and reporting of AOPs as well as use of AOPs to support Integrated Approaches to Testing and Assessment (IATAs) (OECD, 2017b; OECD, 2017a).

The kidney and in particular the proximal tubule epithelium presents one of the key target sites of chemical-induced injury. A wide-range of structurally diverse chemicals, including heavy metals, fungal toxins and drugs are known to cause kidney injury. The particular susceptibility to toxic insult is due to a number of physiological factors that jointly lead to high renal exposure to xenobiotics present in the systemic circulation. The kidneys receive roughly 25% of the cardiac output and hence the rate of delivery of toxicants to the kidney is high. The kidneys’ ability to concentrate solutes further increases exposure of renal cells to xenobiotics (Pfaller and Gstraunthaler, 1998; Khan and Alden, 2002). Uptake of xenobiotics into kidney tubule cells and intrarenal bioactivation to toxic metabolites is facilitated by active transporters and drug metabolizing enzymes that are abundantly expressed particularly throughout the proximal tubule, which renders this segment of the nephron especially susceptible to toxicity (Pfaller and Gstraunthaler, 1998; Khan and Alden, 2002). Acute or chronic damage to proximal tubule cells can lead to kidney dysfunction and ultimately acute or chronic kidney failure. Moreover, chronic cytotoxicity and compensatory regenerative hyperplasia is a well-established mode-of-action by which some chemicals cause kidney tumor formation (Lock and Hard, 2004). Considering the varied chemical nature of nephrotoxic compounds (Pfaller and Gstraunthaler, 1998), it is perhaps not surprising that multiple mechanisms can lead to proximal tubule damage and loss of kidney function. Structurally diverse chemicals may interact with an equally diverse number of molecular targets, and these molecular initiating events (MIE) may each trigger a cascade of molecular and cellular events (Key events, KE) that ultimately result in kidney injury.

In line with recent OECD activities on the use of AOPs in developing Integrated Approaches to Testing and Assessment (IATAs), the overall aim of the present work was to contribute to building a network of AOPs leading to kidney injury through development and critical evaluation of AOPs. Here, we focused on three distinct mechanisms by which certain chemicals or drugs may cause nephrotoxicity and systematically captured the current mechanistic understanding in a formal description of AOPs in accordance with the harmonized terminology provided by the Collaborative Adverse Outcome Pathway Wiki (AOP Wiki; https://aopwiki.org/), a central repository for all AOPs developed as part of the OECD AOP Development Effort. The AOPs considered here are initiated by 1) inhibition of mitochondrial DNA polymerase γ (mtDNA Polγ), 2) receptor mediated endocytosis and lysosomal overload, and 3) covalent protein binding. Human and experimental data on selected chemical stressors for each AOP were identified via Pubmed literatur search and assembled to support the sequence of events leading to kidney injury. The relative level of confidence in the established AOPs was assessed based on evolved Bradford-Hill weight of evidence considerations of biological plausibility, essentiality and empirical support (temporal and dose-response concordance) provided by Becker et al. (2015) and OECD guidance documents for developing and assessing AOPs (OECD, 2017b; 2018) (Box 1).

BOX 1 **Considerations of biological plausibility, essentiality, empirical support, and quantitative and temporal understanding of KERs, including criteria to define the weight of evidence (WoE) (Becker et al., 2015; OECD, 2017b; 2018) [not available in Crossref]**

**Table:** 

Biological plausibility	Biological plausibility is satisfied if a mechanistic link and thus causal relationship between an upstream and downstream KE can be defined that is consistent with the current state of knowledge. WoE is considered high if there is an established mechanistic basis, extensive understanding or broad acceptance. Moderate entails plausibility based on similarity to accepted biological relationships, even though the scientific understanding is incomplete. The level of confidence is low if there is evidence for a statistical association between KE without an understanding of the mechanistic relationship
Essentiality of key events	A key event is considered essential if there is experimental evidence that downstream KEs or the AO will not occur if an upstream event is blocked. This can be achieved e.g. through the use of knockout models or through demonstrating reversibility of an effect (OECD, 2017b). Support for essentiality of KEs is considered high if there is direct evidence from experimental studies demonstrating an impact on downstream KEs if an upstream event is blocked. Moderate support implies indirect evidence for an impact on downstream KEs by modulation of an upstream event. In the absence of any evidence to support essentiality, essentiality is considered low
Empirical evidence: Dose-response and temporal concordance	Dose-response concordance implies that effects on an upstream KE are generally observed at doses of a stressor that are equal or lower than those at which effects on a downstream KE in the AOP are evident. Temporal concordance connotes that the causality chain within an AOP is adequately reflected by the temporal sequence of events that occur within an AOP, i.e. a change in an upstream KEs impacts and thus precedes a downstream KE. WoE for empirical support for KER can be considered high if multiple studies with mutliple stressors demonstrate dependent changes in both KEs. Support is moderate if dependent changes in KEs are shown following exposure to a small number of stressors, with minor inconsistencies that can be explained e.g. by experimental design. Empirical support for KERs is low if there are no or limited studies reporting dependent changes in both KEs or significant inconsistencies
Quantitative and temporal understanding of KERs	For prediction of the state of a downstream KE or the AO based on measurement of an upstream KE, it is essential to understand “how much change in the upstream KE, and/or for how long, is needed to elicit a detectable and defined change in the downstream KE” (OECD, 2018). Information on quantitative relationships may come from studies demonstrating a correlation between two KEs, investigating dose-dependent transitions from one state of a downstream KE to another based on a change in an upstream KE, and defining the response-response relationships through visual presentation, mathematical equations or complex computational modelling approaches (OECD, 2018). The time it takes for a change in an upstream KE to trigger a downstream effect is equally important, particularly when it comes to establishing *in vitro* (or *in vivo*) assays intended to cover different KEs across an AOP.

Considering that implementation of the AOP conceptual framework for translation of mechanistic data into regulatory decisions requires quantitative understanding of the relationships between key events within an AOP (Box 1), information on quantitative relationship between two pairs of KEs - as far as available–is assembled and data gaps that need to be filled in order to move from qualitative descriptions of AOPs to quantitative AOPs are highlighted.

## 2 Inhibition of Mitochondrial Deoxyribonucleic Acid Polymerase γ Leading to Kidney Toxicity (AOP-256)

This Adverse Outcome Pathway describes the sequential key events that link inhibition of mitochondrial DNA polymerase γ (mtDNA Polγ) to kidney toxicity. Nucleoside and nucleotide (nucleos(t)ide) analogs, which are widely used as antiviral drugs for the effective treatment of viral infections, including human immunodeficiency virus (HIV) and chronic hepatitis B virus infections, may act as chemical stressors for this pathway. As structural analogs of substrate nucleotides, these drugs act as chain terminators of viral DNA synthesis via competitive inhibition of reverse transcriptase or viral DNA polymerases, thereby blocking virus replication. Besides targeting viral enzymes, nucleos(t)ide antiviral agents may also interact with human DNA polymerases, which may lead to moderate to life-threatening adverse drug reactions, including peripheral neuropathy, myopathy, lactic acidosis, and acute and chronic kidney injury (Lewis and Dalakas, 1995; Johnson et al., 2001; Fontana, 2009; Fung et al., 2014).

### 2.1 Nephrotoxicity Associated With Long-Term Intake of Acyclic Nucleoside Phosphonates

**FIGURE 1:**
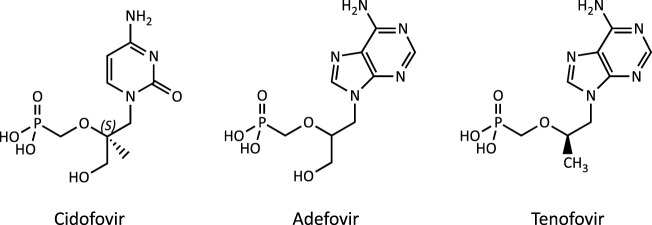
Chemical structure of the acyclic nucleoside phosphonates (ANPs) adefovir, tenofovir and cidofovir.

The acyclic nucleoside phosphonates (ANPs) adefovir, tenofovir and cidofovir (Figure 1) were introduced into drug therapy of viral infections 15–20 years ago. Compared to existing antiviral drugs, this new class of antivirals offered a broad-spectrum activity against DNA viruses and retroviruses and lower risk of resistance development. However, long-term therapy with ANPs was subsequently found to cause renal proximal tubulopathy and even acute kidney injury. Based on its *in vivo* antiretroviral potency (Balzarini et al., 1989), adefovir [9-(2-phosphonylmethoxyethyl)adenine; PMEA] and its prodrug adefovir dipivoxil were originally developed for the treatment of HIV infections and cytomegaly virus (CMV) disease (James, 1997). While initial clinical studies reported effective antiretroviral activity and safety of adefovir dipivoxil (125 mg/d) in patients with advanced HIV infections (Deeks et al., 1997), a subsequent multi-center, randomized, double-blind and placebo-controlled trial in adult patients infected with HIV revealed an increased incidence of nephrotoxic effects in patients receiving adefovir (120 mg/d), characterized primarily by elevations in serum creatinine or hypophosphatemia (Kahn et al., 1999). Considering the risk for serious kidney toxicity in long-term use, the U.S. Food and Drug Administration (FDA) denied approval of adefovir for the treatment of HIV (Highleyman, 1999). However, at lower doses, adefovir dipivoxil was subsequently approved by the FDA for the treatment of hepatitis B, although safety concerns due to nephrotoxicity remain. Tenofovir [9-(2-Phosphonyl-methoxypropyly)adenine; PMPA], respectively its prodrug tenofovir disoproxil fumarate (TDF), obtained FDA approval for the treatment of HIV-1 infections in combination with other antiretroviral medicines in 2002, and subsequently for the treatment of chronic hepatitis B in adults in 2008. While tenofovir is now widely used as a first-line therapy against HIV and hepatitis B virus (HBV) infections, long-term treatment with tenofovir is associated with renal toxicity manifested as proximal tubule dysfunction, renal Fanconi syndrome or even acute kidney injury (Woodward et al., 2009; Agarwala et al., 2010; Herlitz et al., 2010; Gara et al., 2012; Hall, 2013; Sise et al., 2015). Cidofovir [(S)-l-(3-hydroxy-2-phosphonylmethoxypropyl)cytosine, HPMPC] is an acyclic nucleotide analog of deoxycytidine that was approved in 1996 for i.v. treatment of acquired immunodeficiency syndrome (AIDS) associated CMVretinitis in adults. Dose-dependent nephrotoxicity was found to be the major dose-limiting toxicity related to cidofovir treatment, and cases of acute renal failure were reported after treatment with as few as one or two doses (Lalezari et al., 1995; Group, 1997; The Studies of Ocular Complications of AIDS Research Group in collaboration with the AIDS Clinical Trials Group, 2000).

### 2.2 Mechanism of Acyclic Nucleoside Phosphonate Induced Nephrotoxicity

**FIGURE 2:**
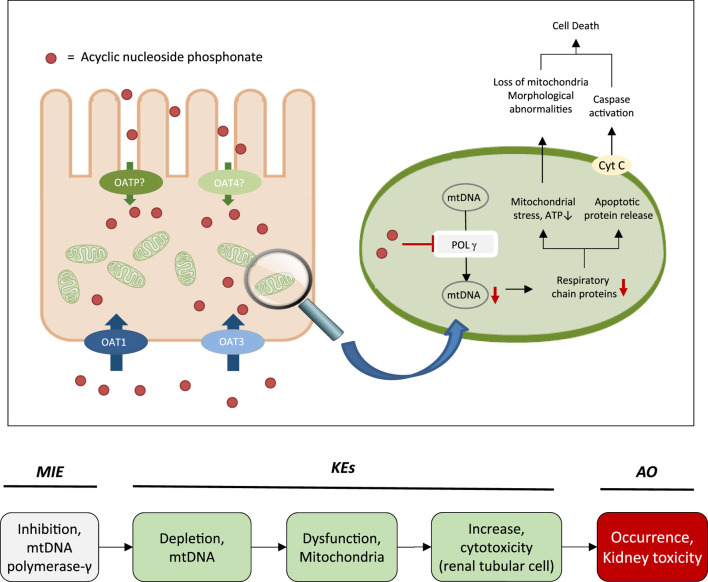
Active transport of acyclic nucleoside phosphonates into proximal tubule cells predominantly via basolateral influx carriers (OATs and OAT3) and subsequent disruption of proximal tubular mitochondrial function due to inhibition of mitochondrial DNA Pol γ (modified from (Fernandez-Fernandez et al., 2011)). Adverse outcome pathway of inhibition of mitochondrial DNA Pol γ leading to kidney toxicity.

While the sequence of molecular events leading to ANP associated nephrotoxicity appears to present a universal mechanism by which nucleos(t)ide analogs may cause toxicity in a wide range of organs and tissues, including liver, heart, muscle and the nervous system (Lewis and Dalakas, 1995; Lewis et al., 2003; Fontana, 2009; Fung et al., 2014), the particular susceptibility of the kidney, respectively the proximal tubule, to ANP toxicity is a result of transporter-mediated uptake into proximal tubule cells, leading to intracellular accumulation of ANPs. Organic anion transporter 1 (OAT1) and to a lesser extent organic anion transporter 3 (OAT3) located at the basolateral membrane of proximal tubule cells are recognized as the major membrane carriers for uptake of ANPs (Figure 2) (Uwai et al., 2007; Zhang et al., 2015; Lash et al., 2018). In support of this, inhibition of basolateral membrane transporters has been shown to reduce ANP nephrotoxicity (Lalezari et al., 1995). In addition, recent data demonstrate ANP uptake into primary human kidney cells via apical carriers, potentially OAT4 or organic anion-transporting polypeptides (OATPs) (Lash et al., 2018). Thus, the tissue-specificity of ANP toxicity appears to be determined predominantly by toxicokinetics and renal handling of these drugs. Once taken up into kidney cells, the phosphonate analogs are transported across the mitochondrial membrane prior or subsequent to metabolic conversion into the active triphosphate form via nucleotide kinases present in mitochondria and the cytosol (Robbins et al., 1995; Lewis et al., 2003; Izzedine et al., 2005; Uwai et al., 2007; Kohler et al., 2011). While designed to inhibit viral reverse transcriptase and DNA polymerases with high efficiency, ANPs may also interact with human DNA polymerases, including mitochondrial DNA Pol γ, which is essential for mitochondrial DNA replication (Figure 2). Phosphorylated ANPs compete with endogenous deoxyribonucleotides for incorporation into DNA, thereby inhibiting mitochondrial DNA Pol γ and consequently mtDNA replication. As a result, mtDNA, which encodes 13 components of the electron transport chain essential to oxidative phosphorylation, is depleted. This leads to impaired mitochondrial function, i.e. reduced respiration, electron leakage and energy decline, and ultimately cell death (Figure 2) (Perazella, 2010; Fernandez-Fernandez et al., 2011). Although additional pathways are discussed as potential contributors to mitochondrial toxicity of nucleos(t)ide analogs (Apostolova et al., 2011), there is sufficient evidence from *in vitro* and *in vivo* studies in humans and rodents to support mitochondrial dysfunction as a consequence of inhibition of mt Pol γ dependent mtDNA replication as the primary mechanism of ANP induced proximal tubule injury.

### 2.3 The Adverse Outcome Pathway of Inhibition of mtDNA Polymerase γ Leading to Kidney Toxicity

Based on these mechanistic considerations, the sequence of key events (KE) leading to kidney injury as an adverse outcome can be described as inhibition of mt Pol γ as the molecular initiating event (MIE), leading to mitochondrial DNA (mtDNA) depletion (KE1), mitochondrial dysfunction (KE2) and proximal tubule cell toxicity (KE3) (Figure 2). Evidence for inhibition of mitochondrial DNA polymerase γ leading to kidney toxicity as an adverse outcome primarily comes from experimental *in vitro* and *in vivo* studies on tenofovir, adefovir and cidofovir that serve as chemical stressors for this pathway, as well as from clinical trials and reports of patients treated with ANPs (Tables 1A–C). Collectively, these studies show a strong association between mitochondrial toxicity and ANP induced nephrotoxicity (Tanji et al., 2001; Cote et al., 2006; Kohler et al., 2009; Lebrecht et al., 2009; Herlitz et al., 2010; Ramamoorthy et al., 2014), with some studies also demonstrating concomitant mtDNA depletion (Tanji et al., 2001; Kohler et al., 2009; Lebrecht et al., 2009; Kohler and Hosseini, 2011). Additional support for mtDNA depletion and mitochondrial dysfunction as down-stream events of mt Pol γ inhibition is derived from studies on nucleos(t)ide analogs that induce mitochondrial toxicity in other target organs via the same principle mechanism. Moreover, there is a wealth of data that link point mutations in the gene encoding for the catalytic subunit of Pol γ with a wide range of human mitochondrial disorders that typically affect tissues with high energy requirement with varying symptoms and severity (Nurminen et al., 2017). In the following sections, evidence supporting the KEs and KE relationships (KERs) in this AOP will be presented, followed by a critical assessment of the AOP in terms of temporal and dose-response concordance, essentiality of key events, biological plausibility, coherence, and consistency of the experimental evidence.

**TABLE 1A:** Evidence from human, animal and *in vitro* studies on tenofovir or its prodrug tenofovir disoproxil fumarate (TDF) supporting the key events and qualitative concordance of KEs within this AOP (n/a = no data available).

		Qualitative concordance		
MIE/KE	Short Name	*In vitro*	Animals	Human
MIE	Inhibition, mt Pol γ	• Inhibition of mtDNA Pol γ by tenofovir demonstrated using purified mtDNA Pol γ (Cherrington et al., 1995) • mtDNA Pol γ mediated incorporation of tenofovir into duplex DNA using recombinant human Pol γ (Johnson et al., 2001; Lee et al., 2003)	n/a	n/a
KE1	Depletion, mtDNA	• Reduced mtDNA levels (1,000 μM; 300 µM n.d.) and reduced expression of proteins involved in mtDNA replication in HK-2 cells and primary mouse renal tubule cells treated with 300 and 1,000 µM TDF for 5 days (Zhao et al., 2017)	• Reduced mtDNA copy numbers in rats treated with TDF (100 mg/kg bw/day) for 8 weeks (Lebrecht et al., 2009). • Decreased mtDNA abundance in proximal tubule isolated by laser-capture microdissection from kidneys of wildtyp and HIV transgenic mice treated with TDF (5 mg/kg bw) for 5 weeks (Kohler et al., 2009; Kohler and Hosseini, 2011). • Down-regulation of proteins involved in mtDNA replication in mice treated with TDF (10 mg/kg bw) for 8 weeks (Zhao et al., 2017)	n/a
KE2	Dysfunction, Mitochondria	• Irregular shaped mitochondria and disrupted cristae, reduced mitochondria membrane potential (MMP), respiration and ATP in HK-2 cells treated with 300 and 1,000 µM TDF (Zhao et al., 2017)	• Enlarged mitochondria with disrupted crystal architecture and dysfunction of mtDNA-encoded respiratory chain subunits in rats treated with TDF (100 mg/kg bw/day) for 8 weeks (Lebrecht et al., 2009). • Increased mitochondrial number, irregular mitochondrial shape with fragmented cristae in kidneys of wildtyp and HIV transgenic mice treated with TDF (5 mg/kg bw) for 5 weeks (Kohler et al., 2009; Kohler and Hosseini, 2011). • Renal mitochondrial dysfunction (measured as respiratory control ratio, MTT reduction, and mitochondrial swelling) and reduced activity of ETC complexes I, II, IV, and V in rats treated with 600 mg/kg body weight TDF for 5 weeks (Ramamoorthy et al., 2014)	• Mitochondrial enlargement, depletion, and dysmorphic changes in HIV patients treated with TDF (Herlitz et al., 2010)
KE3	Increase, Cytotoxicity	• Cytotoxicity of tenofovir in a range of kidney cell models with the severity depending on cellular uptake (Zhang et al., 2015; Nieskens et al., 2016). • Loss of cell viability in HK-2 and primary mouse renal tubule cells treated with 300 and 1,000 µM TDF for 5 days (Zhao et al., 2017)	• Dilated proximal tubules in rats treated with TDF (100 mg/kg bw/day) for 8 weeks (Lebrecht et al., 2009). • No detectable renal tubular damage in renal cortex of wildtyp and HIV transgenic mice treated with TDF (5 mg/kg bw) for 5 weeks (Kohler et al., 2009; Kohler and Hosseini, 2011). • Loss of tubular cells and tubular vacuolization in mice treated with TDF (10 mg/kg bw) for 8 weeks (Zhao et al., 2017). • Distorted proximal convoluted tubules with destroyed lining epithelium, epithelial desquamation, mild necrotic change in rats treated with TDF (600 mg/kg bw) for 5 weeks (Ramamoorthy et al., 2014)	• Acute tubular necrosis in proximal tubules in HIV patients treated with TDF (Herlitz et al., 2010)
AO	Occurrence, Kidney Toxicity	–	• Decrease in kidney weight in rats treated with TDF (100 mg/kg bw/day) for 8 weeks (Lebrecht et al., 2009). • Impaired tubular function evidenced by tubular proteinuria, increased urinary phosphate, potassium, and bicarbonate excretion and a considerable reduction in serum phosphate and potassium in rats treated with TDF (600 mg/kg bw) for 5 weeks (Ramamoorthy et al., 2014). • Reduced creatinine clearance, increased sCrea and BUN in mice treated with TDF (10 mg/kg bw) for 8 weeks (Zhao et al., 2017). • Dose-dependent decrease in creatinine clearance and increased renal vascular resistance in rats fed diets containing TDF (50 and 300 mg TDF/kg food) for 30 days (Liborio et al., 2008)	• Proteinuria, glycosuria, increases serum creatinine in HIV patients treated with TDF; Recovery of renal function after TDF discontinuation (Herlitz et al., 2010). • Proteinuria and progressive renal dysfunction in a HIV patient (TDF + co-medication) (Agarwala et al., 2010). • Tubular proteinuria, reduced tubular transport maximum of phosphate, glycosuria, increased sCrea and reduced estimated GFR in HIV patients treated with TDF, with partial recovery after discontinuation of treatment (Woodward et al., 2009)

**TABLE 1B:** Evidence from human, animal and *in vitro* studies on **adefovir** or its prodrug adefovir dipivoxil (ADV) supporting the key events and qualitative concordance of KEs within this AOP (n/a = no data available).

		Qualitative concordance		
MIE/KE	Short Name	*In vitro*	Animals	Human
KE1	Depletion, mtDNA	• Reduced mtDNA levels (1,000 μM; 300 µM n.d.) and reduced expression of proteins involved in mtDNA replication in HK-2 cells and primary mouse renal tubule cells treated with adefovir (300 and 1,000 µM) for 5 days (Zhao et al., 2017)	• Reduced expression of proteins involved in mtDNA replication in mice treated with adefovir dipivoxil (10 mg/kg bw) for 8 weeks (Zhao et al., 2017)	• Reduced mtDNA content in proximal tubule cells of an HIV-infected individual treated with ADV and other antivirals) for 7 months (Tanji et al., 2001)
KE2	Dysfunction, Mitochondria	• Irregular shaped mitochondria and disrupted cristae, reduced mitochondria membrane potential (MMP), respiration and ATP in HK-2 cells treated with 300 and 1,000 µM adefovir for 5 days (Zhao et al., 2017)	n/a	• Mitochondrial alterations accompanied by decreased cyclooxygenase (COX) activity in an HIV-infected individual treated with ADV and other antivirals) for 7 months (Tanji et al., 2001)
KE3	Increase, Cytotoxicity	• Loss of cell viability in HK-2 and primary mouse renal tubule cells treated with 300 and 1,000 µM adefovir for 5 days (Zhao et al., 2017) • Cytotoxicity of adefovir in HEK293 cells transfected with organic anion transporter (OAT) 1 and 3 (Zhang et al., 2015)	• Loss of tubular cells and tubular vacuolization in mice treated with ADV (10 mg/kg bw) for 8 weeks (Zhao et al., 2017)	• Severe acute degenerative changes in proximal tubules of an HIV-infected individual treated with ADV (and other antivirals) for 7 months (Tanji et al., 2001) • Acute tubular necrosis in a renal transplant recipient with Hepatitis B virus infection treated with ADV 10 mg daily (+co-treatment with immunosuppressants) (Izzedine et al., 2009). • Mild renal tubular atrophy, interstitial fibrosis and atherosclerosis in a patient treated with ADV for the treatment of chronic hepatitis B virus (HBV) (Lin et al., 2017)
AO	Occurrence, Kidney Toxicity	–	• Reduced creatinine clearance, increased sCrea and BUN in mice treated with ADV (10 mg/kg bw) for 8 weeks (Zhao et al., 2017)	• Renal failure in an HIV-infected individual treated with ADV (and other antivirals) for 7 months (Tanji et al., 2001) • Acute renal failure in a renal transplant recipient with Hepatitis B virus infection treated with ADV 10 mg daily (+co-treatment with immunosuppressants) (Izzedine et al., 2009) • Elevations in serum creatinine or hypophosphatemia in 60% of patients treated with ADV (120 mg/d) for 24 weeks, usually returning to baseline after discontinuation of adefovir (Kahn et al., 1999) • Serum creatinine increase, hypophosphatemia and proteinuria in patients with chronic hepatitis B receiving ADV 30 mg/d for a median duration of 48 weeks, no or minimal effects at a dose of 10 mg/d (Izzedine et al., 2004) • Fanconi syndrome characterized by hypophosphatemia, elevated sCr, reduced GFR positive urinary protein, erythrocytes and glucose in 28 patients treated with ADV for the treatment of chronic hepatitis B virus (HBV) (Lin et al., 2017) • Renal tubular dysfunction characterized by increased sCr and/or hypophosphatemia, hypouricaemia and mild proteinuria in 15% of patients treated with adefovir for 2–9 years, partial reversibility with change to other antivirals (Gara et al., 2012)

**TABLE 1C:** Evidence from human, animal and *in vitro* studies on **cidofovir** supporting the key events and qualitative concordance of KEs within this AOP (n/a = no data available).

		Qualitative concordance		
MIE/KE	Short Name	*In vitro*	Animals	Human
KE1	Depletion, mtDNA	n/a	n/a	n/a
KE2	Dysfunction, Mitochondria	n/a		• Diffuse mitochondrial swelling to profound morphologic mitochondrial changes in kidney biopsies of patients receiving 2.5 mg/kg cidofovir (Talmon et al., 2010)
KE3	Increase, Cytotoxicity	• Cytotoxicity of cidofovir in HEK293 cells transfected with organic anion transporter (OAT) 1 and 3 (Zhang et al., 2015)	• Degenerative changes characterized by tubular depletion and degeneration, tubular cytomegaly, tubular karyomegaly, and tubular regeneration in the outer cortex of rats treated with cidofovir (100 mg/kg bw) for 5 days (Bischofberger et al., 1994)	n/a
AO	Occurrence, Kidney Toxicity	n/a	• Degenerative changes characterized by tubular depletion and degeneration, tubular cytomegaly, tubular karyomegaly, and tubular regeneration in the outer cortex of rats treated with cidofovir (100 mg/kg bw) for 5 days (Bischofberger et al., 1994)	• Acute kidney injury (defined as increase in sCr level of ≥0.3 mg/dl from baseline) in pediatric patients receiving cidofovir for the treatment of adenovirus infection (Vora et al., 2017)

#### Molecular Initiating Event: Inhibition of mtDNA Polymerase γ

As structural analogs of normal nucleotides that lack the 3′-OH group of the deoxyribose moiety, antiviral nucleos(t)ides were designed as alternative substrates for viral DNA polymerases that block virus replication by preventing chain-elongation. As an undesirable extension of their pharmacological action, antiviral nucleos(t)ides also interact with host DNA polymerases. Among the cellular replicative DNA polymerases, mitochondrial DNA Pol γ, which is responsible for maintenance of mtDNA, has been shown to be most sensitive to the inhibitory effects of these drugs, although nuclear DNA polymerases such as DNA polymerase α and β may also be affected. Numerous *in vitro* and *in vivo* studies document inhibitory effects of a wide range of nucleoside and nucleotide reverse transcriptase inhibitors on mtDNA Pol γ at concentrations achieved *in vivo* (reviewed in (Kakuda, 2000)). While there are significant differences in the ability of individual antiviral nucleos(t)ides to become incorporated into DNA by Pol γ, quantitative prediction of the overall inhibitory effect on mtDNA replication and subsequent mitochondrial toxicity is complicated by the 3′-5′exonuclease activity of Pol γ, which catalyses removal of incorporated nucleotides. This is exemplified by the case of zidovudine (3′-azido-3′-deoxythymidine), a drug that is a comparatively poor substrate for incorporation into mtDNA by Pol γ, which may still effectively block mtDNA replication due to inefficient excision of dideoxynucleotides and hence persistence in mtDNA (Lim and Copeland, 2001; Lim et al., 2003). In addition to the intrinsic 3′-5′exonuclease activity of Pol γ, a recent study also identified Pol β, previously thought to be exclusively located in the nucleus, as a major mtDNA repair enzyme (Prasad et al., 2017). Thus, the ability of nucleos(t)ide analogs to inhibit Pol β presents a further modifying events in this pathway, with progression to the next key event, i.e. mtDNA depletion, depending on the rate of nucleotide incorporation vs. the rate of removal by Pol γ (and presumably also by Pol β) relative to the time required to replicate mtDNA.

Using purified mtDNA Pol γ and activated calf thymus DNA as a primer template, adefovir, tenofovir and cidofovir were all shown to inhibit mammalian DNA polymerases α, β, and γ (Tables 1A–C). The kinetic inhibition constants (K_i_) values of the diphosphates of the three nucleoside phosphonates against Pol γ were 0.97, 59.5 and 299 µM for adefovir, tenofovir and cidofovir, respectively, and with the exception of adefovir - significantly higher than K_i_ values of some of the other nucleos(t)ide analogs such as 2′,3′-dideoxycytidine (0.034 µM) or zidovudine (18.3 µM) (Cherrington et al., 1994; Cherrington et al., 1995). The kinetic inhibition constants against mammalian DNA polymerases β were 70.4, 81.7 and 520 µM for adefovir, tenofovir and cidofovir, respectively (Cherrington et al., 1994; Cherrington et al., 1995). The lower inhibitory activity of tenofovir and cidofovir against human DNA polymerases compared to adefovir and some of the other antiviral nucleos(t)ide analogs were considered to be in line with the relatively lower toxicity of tenofovir and cidofovir. Similarly, a toxicity index calculated based on single turnover kinetic studies using reconstituted human Pol γ holoenzyme to measure the rates of incorporation and exonuclease removal also suggested relatively low mitochondrial toxicity of tenofovir as compared to some other drugs, e.g., 2′,3′-dideoxycytidine (Johnson et al., 2001; Lee et al., 2003).

The inhibitory effects of antiviral drugs on Pol γ dependent mtDNA replication resemble mitochondrial genetic diseases associated with inactivating mutations in the gene encoding Pol γ. Pathogenic mutations in the catalytic subunit of Pol γ cluster into five distinct regions involving the active site, residues of the upstream DNA binding channel, and regions responsible for regulating polymerase vs. exonuclease activity and enzyme processivity (Nurminen et al., 2017). The clinical manifestations of Pol γ syndromes comprise a continuum of phenotypic abnormalities with varying degree of severity, age of onset and tissues affected. There is a close relationship between the age of onset and the severity of the symptoms, i.e. the earlier the onset, the more severe the condition. These range from prenatally-fatal to severe early childhood multi-system disorders such as Alpers-Huttenlocher syndrome (AHS), a progressive neurodegenerative disorder accompanied by disturbed hepatocellular function and tissue-specific DNA depletion (liver > skeletal muscle, heart) that progressively leads to psychomotor regression, epilepsy and liver failure, to adult-onset milder diseases such as progressive external ophthalmoplegia (Cohen et al., 1993; Nurminen et al., 2017). The latter initially presents with weakness of the eye muscles but may also involve other multisystemic features including generalized mitochondrial myopathy with ragged-red fibers, ataxia, axonal sensory-motor polyneuropathy, sensorineural hearing loss, depression, and lactic acidosis (Cohen et al., 1993). Childhood myocerebrohepatopathy spectrum (MCHS) is another Pol γ-related disorder that presents in the first few months of life with developmental delay, lactic acidosis, myopathy and further symptoms such as frequent vomiting, hearing loss, liver failure, pancreatitis and renal tubular acidosis (Cohen et al., 1993).

#### Key Event 1: mtDNA Depletion

As Pol γ is essential for mtDNA replication, a gradual decrease in mtDNA is an obvious and biologically plausible consequence of sustained inhibition of Pol γ. While there are no reports on the effect of cidofovir on mtDNA content (Table 1C), experimental *in vitro* and *in vivo* studies demonstrate reduced mtDNA copy numbers associated with decreased expression of proteins involved in mtDNA replication in kidney tubule cells in response to adefovir and tenofovir (Tables 1A, B). A reduction in the ratio of mitochondrial to nuclear DNA was also reported in proximal tubule cells of an HIV-infected individual maintained on highly active antiretroviral therapy that included adefovir dipivoxil for 7 months (Tanji et al., 2001). In contrast to these studies, Birkus et al. found no effect of tenofovir on mtDNA content in HepG2 cells, skeletal muscle cells and human renal proximal tubule epithelial cells (Birkus et al., 2002). It needs to be emphasized, however, that uptake of tenofovir into cells was not verified in this study, either by directly measuring intracellular levels of tenofovir or by characterization of cells with regard to expression of relevant drug transporters. Considering that tenofovir toxicity depends on transporters that mediate cellular uptake (Uwai et al., 2007; Zhang et al., 2015; Lash et al., 2018), it is questionable if sufficiently high intracellular concentrations to inhibit Pol γ and block mtDNA replication were achieved in this model.

The causal relationship between inhibition of Pol γ and loss of mtDNA is further supported by studies investigating the mechanism of toxicity of nucleos(t)ide analogs in other cells and tissues. For instance, a significant reduction in mtDNA was observed in muscle biopsies of zidovudine-treated HIV positive patients with myopathy as compared non-HIV-patient controls (Arnaudo et al., 1991). Inhibition of mtDNA synthesis and loss of cell number was also observed in a T-lymphoid leukemic cell line (Molt-4) treated with several anti-HIV and anti-HBV nucleoside analogs (d4T, 3′-deoxy-2′,3′-didehydrothymidine; FLT, 3′-fluoro-3′-deoxythynidine; ddC, 2′,3′-dideoxycytidine), which were also identified as potent inhibitors of Pol γ. Similar to the study on tenofovir by Birkus et al. (Birkus et al., 2002), a number of potent Pol γ inhibitors did not cause significant effects on mtDNA synthesis and cell viability (Martin et al., 1994). Based on these findings, the authors concluded that there was no clear quantitative or qualitative correlation between the inhibition of isolated Pol γ and inhibition of mitochondrial DNA synthesis *in vitro*, and moreover that these data are not predictive of *in vivo* toxicity (Martin et al., 1994). It is however important to stress that toxicokinetics, most notably cellular uptake of the tested antivirals, were not considered in this assessment. Thus, it is possible that some of the most potent inhibitors of Pol γ identified in a cell-free assay failed to induce mtDNA depletion and cytotoxicity in this cell model simply because of insufficient cellular uptake (Martin et al., 1994).

Experimental evidence for functional inhibition of mitochondrial Pol γ as the underlying cause of mtDNA depletion and associated mitochondriopathies also comes from animal studies. Functional knockout of Pol γ in mice leads to complete loss of mtDNA and embryonic lethality in mice (Hance et al., 2005). Similarly, Pol γ function was demonstrated to be essential for maintenance of mtDNA and development in *Drosophila melanogaster* (Iyengar et al., 2002). In contrast, polg−/− mutant zebrafish carrying mutations within the polymerase domain survived up to 4 weeks post-fertilization, but showed delayed growth and regenerative defects accompanied by a gradual decrease in mtDNA that correlated with impaired basal and maximal FCCP (carbonyl cyanide p-trifluoro-methoxyphenyl hydrazone)-uncoupled respiration (Rahn et al., 2015). This study also revealed tissue specific differences in the basal levels of mtDNA copy numbers per cell in wildtype animals, with the tail region of zebrafish containing higher levels of mtDNA compared to the region containing the gills, heart and internal organs, and yet lower levels in the central nervous system (CNS) region containing eyes and the brain. Moreover, the degree of mtDNA depletion upon Pol γ knockout was shown to differ between tissues, with the tissue most severely depleted being the organ fraction (mtDNA content in polg−/− 14% of wildtype), followed by CNS (38% of wildtype) and finally tail as the least affected region (52% of wildtype) (Rahn et al., 2015). Considering the finding that the organ fraction, which contains e.g. the liver, along with CNS were most affected by Pol γ knockout, it was suggested that the polg−/−zebrafish model closely resembles human Pol γ-associated mitochondrial diseases that typically present with first symptoms in organs with high energy demand, i.e., CNS and the liver.

Differences in the rate of mtDNA synthesis between tissues are also likely to be an important determinant of tissue-specific responses to Pol γ inhibition. *In vivo* mtDNA labeling with BrdU in adult wild-type mice showed that BrdU was more rapidly incorporated into mtDNA in the brain as compared to the liver (Fuke et al., 2014), suggesting more rapid mitochondrial biogenesis in the brain.

#### Key Event 2: Mitochondrial Dysfunction

Although the vast majority of proteins localized in mitochondria is encoded by nuclear DNA, the mitochondrial genome is essential for oxidative energy metabolism as all 13 polypeptides coded for by mitochondrial genes are subunits of complexes of the respiratory chain/oxidative phosphorylation system. It is therefore inevitable that depletion of mtDNA leads to mitochondrial dysfunction. Evidence for mitochondrial dysfunction as a key event in this AOP comes from human, animal and *in vitro* studies treated with ANPs as chemical stressors for this pathway (Tables 1A–C). Mitochondrial changes indicative of impaired mitochondrial function are typically described as mitochondrial enlargement with disrupted crystal architecture and reduced activity of mtDNA-encoded respiratory chain subunits. These alterations were frequently reported to occur concomitant with mtDNA depletion and cytotoxicity, further supporting a direct link between upstream and downstream key events (Kohler et al., 2009; Lebrecht et al., 2009; Kohler and Hosseini, 2011; Zhao et al., 2017). Likewise, studies in Pol γ deficient animal models demonstrate a close correlation between loss of mtDNA induced by Pol γ inactivation and altered mitochondrial function (e.g. impaired basal and maximal FCCP-uncoupled respiration (Rahn et al., 2015)). Although genetic knockdown of Pol γ typically affects different tissues than ANPs which specifically target the kidney in a transporter-dependent manner, these studies provide substantial evidence for a causal relationship between mtDNA depletion and mitochondrial dysfunction.

#### Key Event 3: Cytotoxicity

Mitochondria are not only critical for cellular metabolism and energy production that are fundamental to cell viability, they also act as signalling organelles and as such play a key role in cellular life-and-death decisions. Mitochondria participate in both the extrinsic and intrinsic pathway of apoptosis, the latter of which involves opening of the mitochondrial outer membrane and subsequent release of pro-apoptotic factors such as cytochrome C from mitochondria. Interference with the energy-producing function of mitochondria, e.g. through impairment of oxidative phosphorylation as a result of decreased mtDNA content, leads to adenosine triphosphate (ATP) depletion and consequently disturbed cellular function that culminates in necrosis.

While there are multiple mechanisms by which drugs and chemicals can target mitochondria and impair mitochondrial ATP synthesis (e.g. uncoupling of the mitochondrial respiratory chain, inhibition of ATP synthesis, damage to mtDNA, interference with mtDNA replication), mitochondrial toxicity is well established as a key cause of toxicity of a wide range of drugs and chemicals that affect different target organs, including the liver, heart, skeletal muscle, central nervous system, and the kidney. Similarly, mitochondrial dysfunction caused by inherited or sporadic mutations in mtDNA is considered to play a critical role in the pathogenesis of a range of diseases, including acute and chronic kidney injury that involve damage to the proximal tubule (Martin-Hernandez et al., 2005; Emma et al., 2012; Che et al., 2014; Hall and Schuh, 2016). Within the kidney, tubule cells and particularly those of the proximal tubules, are particularly vulnerable to mitochondrial dysfunction. Active transport of solutes in the proximal tubule requires large amounts of ATP generated predominantly via mitochondrial oxidative phosphorylation. To meet the high energy demand, proximal tubule cells contain numerous large mitochondria. While it is generally acknowledged that mitochondrial dysfunction may lead to activation of cell-death pathways, evidence for a mechanistic link between mitochondrial dysfunction caused by inhibition of mtDNA Pol γ and proximal tubule toxicity comes from *in vitro* and *in vivo* studies that demonstrate loss of cell viability, dilated proximal tubules and degenerative changes affecting proximal tubules in experimental animals and humans treated with ANPs (Tables 1A–C).

#### Adverse Outcome: Kidney Toxicity

Through excretion of metabolic wastes and regulation of acid-base balance, electrolyte concentrations and extracellular fluid volume, the kidney plays a key role in maintaining whole-body homeostasis. Functional integrity of the proximal tubule, which contributes to fluid, electrolyte, and nutrient homeostasis by reabsorbing approximately 60–80% of filtered solute and water as well as virtually all of the filtered nutrients (e.g., glucose and amino acids) and low-molecular-weight proteins, is critical for whole-kidney function. Consequently, injury to the proximal tubule will lead to a decline in kidney function, although minor proximal tubule changes may not cause significant effects on renal function due to the kidney’s functional reserve and capacity to regenerate. Numerous drugs and chemicals are known to cause nephrotoxicity primarily by killing proximal tubule cells. Depending on the nature and severity of the insult, altered tubule or whole kidney function may be evidenced by altered renal handling of electrolytes (e.g., sodium, phosphate, calcium, bicarbonate), an increase in urinary glucose, amino acids and low-molecular-weight proteins indicative of impaired tubular reabsorption, and a rise in blood urea nitrogen (BUN) and serum creatinine (sCr). Such changes are evident in experimental animals and patients treated with ANPs that act as chemical stressors for this AOP (Tables 1A–C). For instance, tubular proteinuria and increased urinary phosphate, potassium, and bicarbonate excretion accompanied by reduced serum phosphate and potassium were observed in rats treated with TDF at 600 mg/kg bw for 5 weeks (Ramamoorthy et al., 2014). In another study in rats, a dose-dependent decrease in creatinine clearance was observed (Liborio et al., 2008). Reduced creatinine clearance accompanied by increased sCr and BUN were also reported in mice treated with TDF and adefovir dipivoxil (Zhao et al., 2017). In humans, kidney toxicity associated with intake of ANPs is predominantly characterized by glucosuria and proteinuria, hypophosphatemia, and increased sCr (Kahn et al., 1999; Izzedine et al., 2004; Woodward et al., 2009; Agarwala et al., 2010; Herlitz et al., 2010; Gara et al., 2012; Lin et al., 2017; Vora et al., 2017).

### 2.4 Assessment of the Adverse Outcome Pathway of Inhibition of mtDNA Polymerase γ Leading to Kidney Toxicity

The relative level of confidence in the overall AOP was assessed based on evolved Bradford-Hill weight of evidence considerations provided by Becker et al. (2015) and OECD guidance documents for developing and assessing AOPs (OECD, 2017b; OECD, 2018) (Box 1).

#### Biological Plausibility

Mitochondrial DNA replication relies on Pol γ activity. As detailed in Section 2.3, sustained inhibition of Pol γ inevitably leads to reduced mtDNA synthesis and in consequence to a gradual decrease in mtDNA. Loss of mtDNA is thus an obvious and biologically plausible consequence of inhibition of Pol γ (Table 2). All 13 polypeptides encoded by mtDNA are subunits of complexes of the respiratory chain/oxidative phosphorylation system that are required for maintaining mitochondrial function. Biological plausibility for the KER between depletion of mtDNA and mitochondrial dysfunction is therefore considered high (Table 2). It is also well established that mitochondrial function is vital for cell survival, particularly in cells with a high energy demand such as proximal tubule cells. Finally, it is well established and supported by an extensive body of evidence that proximal tubule cell injury impairs kidney function. Thus, the level of confidence in the biological plausibility of all key event relationships (KERs) within the proposed AOP can be considered as high (Table 3).

**TABLE 2:** Dose-Time Concordance of KEs based on rodent studies with tenofovir disoproxil fumarate (TDF) as a specific stressor for the adverse outcome pathway of inhibition of mitochondrial DNA polymerase γ leading to kidney toxicity (n.d. = not determined; n/a = no data available).

	Temporal concordance				
**Dose-response concordance**	Species	Dose (mg/kg bw)	5 weeks	8 weeks	References
	Mice	5	KE1 mtDNA ↓ KE2 mt dysfunction KE3 - AO -	n/a	(Kohler et al., 2009; Kohler and Hosseini, 2011)
		10	n/a	KE1 *n.d.* (but effect on mtDNA replication machinery) KE2 n.d KE3 PT injury AO Kidney toxicity	Zhao et al. (2017)
	Rats	100	n/a	KE1 mtDNA ↓ KE2 mt dysfunction KE3 PT injury AO Kidney toxicity	Lebrecht et al. (2009)
		600	KE1 *n.d* KE2 mt dysfunction KE3 PT injury AO Kidney toxicity	n/a	Ramamoorthy et al. (2014)

**TABLE 3:** Weight-of-evidence analysis of KERs in the adverse outcome pathway of inhibition of mitochondrial DNA polymerase γ leading to kidney toxicity.

KE Upstream	KE Downstream	Weight of evidence (WoE) for KER			
		Biological Plausibility	Essentiality	Empirical support	Overall WoE
Inhibition, mt Pol γ	Depletion, mtDNA	high	high	moderate	high
Depletion, mtDNA	Dysfunction, Mitochondria	high	high	moderate	high
Dysfunction, Mitochondria	Increase, Cytotoxicity	high	high	high	high
Increase, Cytotoxicity	Occurrence, Kidney Toxicity	high	high	high	high

#### Essentiality of Key Events

While there are numerous studies to demonstrate that blocking Pol γ function via pharmacological inhibition, genetic knock-out or mutational inactivation is detrimental to cells as it leads to mtDNA depletion and reduced mitochondrial function, there do not appear to be any experimental studies to investigate if restoration of Pol γ function (e.g., through overexpression of Pol γ) maintains mtDNA copy numbers and mitochondrial function. However, studies on arterial aging in mice show that restoring mtDNA copy numbers through overexpression of the mitochondrial helicase Twinkle (Tw^+^) preserves arterial mitochondrial respiration in aging mice (Foote et al., 2018). Similarly, in a mouse model of volume overload-induced heart failure, increased mtDNA copy numbers in transgenic mice overexpressing human transcription factor A of mitochondria (TFAM) or Twinkle helicase afforded cardioprotection through maintaining mitochondrial enzymatic activities (Ikeda et al., 2015). These data provide experimental support for the essentiality of mtDNA copy number for mitochondrial respiration. Maintaining mitochondrial function has been recognized as a promising therapeutic target for the treatment of acute kidney injury (Hall and Schuh, 2016). Strategies to increase mitochondrial biogenesis, e.g., through activation or overexpression of peroxisome proliferator-activated receptor-γ coactivator-1α (PGC-1α) that acts as a master regulator of mitochondrial biogenesis, have been demonstrated to restore mitochondrial activity and/or kidney function in ischemia-reperfusion induced renal injury or drug-induced acute kidney injury (Sivarajah et al., 2003; Rasbach and Schnellmann, 2007; Liborio et al., 2008; Funk and Schnellmann, 2013; Jesinkey et al., 2014; Jesse et al., 2014). Collectively, these studies provide good evidence that mitochondrial dysfunction and kidney toxicity as the adverse outcome can be prevented by maintaining mtDNA levels, which requires mitochondrial biogenesis and hence Pol γ mediated mtDNA replication. Moreover, recovery of kidney function was reported in patients after discontinuation of ANP treatment (Kahn et al., 1999; Woodward et al., 2009; Herlitz et al., 2010; Gara et al., 2012). Based on direct evidence illustrating essentiality for at least one of the important KEs, the level of confidence for essentiality of KEs in this AOP can thus be considered as high (Table 2).

#### Empirical Evidence: Dose-Response and Temporal Concordance

Overall, there is only very limited data on dose-related effects of ANPs *in vitro* and *in vivo*. In HK-2 cells and primary mouse renal tubule cells treated with TDF and ADV at 300 and 1,000 µM for 5 days, irregularly shaped mitochondria accompanied by reduced mitochondrial respiration and ATP production were observed at both concentrations (Zhao et al., 2017). While treatment with 1,000 µM TDF and ADV resulted significant inhibition of cell growth, decrease in cell viability and induction of apoptosis, treatment with 300 µM of each ANP did not significantly affect cell viability (Zhao et al., 2017). These data provide evidence that mitochondrial dysfunction (KE2) occurs at equal and lower concentrations of adefovir and tenofovir than cytotoxicity (KE3). Unfortunately, mtDNA copy number and expression of proteins involved in mtDNA replication were only investigated at 1,000 µM of TDF and ADV, at which they were significantly altered. Although these data show that all KE were impacted at the same concentration, it is not possible to conclude that KE1 (mtDNA depletion) occurs at lower concentration than the downstream KEs. There are no *in vivo* studies investigating dose-related effects of ANPs on the proposed KEs, i.e., all studies conducted in experimental animals so far are limited to a single dose group per study (Table 3). Cross-study comparison to establish dose-response concordance is not possible due to variations in experimental design, including species, strain, dose and treatment duration. In studies in mice given TDF at a dose of 5 mg/kg bw for 5 weeks, loss of mtDNA and mitochondrial dysfunction were observed in the absence of proximal tubule injury and impaired kidney function (Kohler et al., 2009; Kohler and Hosseini, 2011), suggesting that either higher doses or prolonged treatment may be required to trigger the final KE and the AO in this AOP (Table 3). In another study, effects on mtDNA replication machinery, proximal tubule injury and kidney toxicity were evident in mice given TDF at 10 mg/kg bw for 8 weeks, while mitochondrial function was not assessed (Table 3) (Zhao et al., 2017). In rats, all 3 KE and kidney toxicity as the adverse outcome were observed after treatment with TDF (100 mg/kg bw) for 8 weeks (Table 3) (Lebrecht et al., 2009). Mitochondrial dysfunction, proximal tubule injury and kidney toxicity were also evident in rats given TDF at 600 mg/kg bw for 5 weeks, but mtDNA copy number was not assessed in this study (Table 3) (Ramamoorthy et al., 2014). Collectively, the available *in vitro* and *in vivo* studies conducted using chemical stressors for this AOP generally demonstrate effects on KEs across the entire AOP at equal doses/concentrations of the stressor, with some evidence for upstream events occurring at lower concentrations than downstream KEs. There are no data that would disagree with the assumption of dose-response concordance.

As can be seen from Table 3, there are no *in vivo* time-course studies on tenofovir or other specific stressors for this AOP, and hence temporal concordance for the entire sequence of events cannot be demonstrated. However, there is a large body of evidence to demonstrate that changes in mitochondrial bioenergetics and dynamics precede proximal tubule damage in kidney injury induced by nephrotoxic drugs and chemicals (Lock et al., 1993) as well as in diabetic kidney disease (Coughlan et al., 2016).

Based on the criteria for assessing AOP (Box 1), evidence for dose-response concordance with relevant stressors but lack of time-course studies to demonstrate temporal concordance, the level of confidence for empirical support for the KERs in this AOP is considered moderate (Table 2).

#### Weight-Of-Evidence Analysis

Based on the high level of confidence in the biological plausibility of KERs, strong support for essentality of the KEs provided i.e. by experimental studies demonstrating mtDNA depletion and reduced mitochondrial function in response to genetic knock-out or mutational inactivation of Pol γ, and moderate empirical support for the KER in this AOP, the overall weight-of-evidence of this AOP can be considered as high (Table 2).

### 2.5 Quantitative and Temporal Understanding of Key Event Relationships

Based on the available literature, there is at present little or no quantitative information on the response-response relationship between two pairs of KEs in the AOP of inhibition of Pol γ leading to depletion of mtDNA, but experiments are underway within the Risk-IT project to define these.

#### KER1: Inhibition of Pol γ Leading to Depletion of mtDNA

**FIGURE 3:**
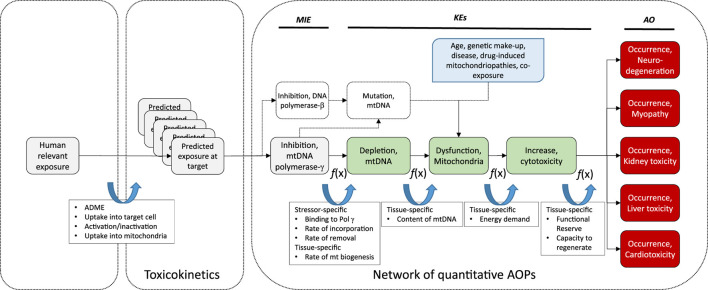
Schematic presentation of a universal AOP of Pol γ inhibition leading to adverse outcomes in kidney and extrarenal tissues, with predicted exposure at target site, feed-forward and feedback mechanisms, and potential modulating factors as determinants of the tissue-specific biological response to a chemical stressor.

As outlined above, the quantitative relationship between inhibition of Pol γ and mtDNA depletion is still poorly defined. Efforts to predict a compounds inhibitory effect on mtDNA replication based on its inhibitory activity against Pol γ did not yield satisfactory results. In establishing response-response relationships, several aspects that determine the KER need to be considered. Firstly, inhibitory effects on Pol γ are typically assessed in cell-free systems, whereas studying effects on mtDNA replication require intact cells. Nominal concentrations added to a cell culture system may not adequately reflect concentrations at the molecular target, e.g., due to active transport, drug metabolism, or binding to plastic. Thus, adequate understanding of the *in vitro* toxicokinetics of the chemical stressors is needed to extrapolate from a cell-free to a cell-based assay. Second, the overall effect of a chemical stressor on Pol γ mediated mtDNA replication as the downstream KEs depends on its persistence in mtDNA, which is a function not only of the stressors ability to bind to Pol γ and become incorporated into mtDNA (feed-forward loops), but also on the rate of excision of nucleotides by the intrinsic proofreading 3′-5′exonuclease activity of Pol γ (Johnson et al., 2001; Lim and Copeland, 2001; Lim et al., 2003), which presents a feed-back loop (Figure 3). Moreover, recent evidence suggest that Pol β also contributes to base excision repair in mammalian mitochondria (Prasad et al., 2017). Thus, the ability of a chemical stressor to inhibit Pol β is also likely to influence its persistence in mtDNA and thus its overall effect on mtDNA replication. Finally, the rate of mitochondrial biogenesis is a critical determinant. If the stressor is removed more quickly from mtDNA than is required for mtDNA to replicate, mtDNA copy number may not be affected. Similarly, exposure to a stressor for only a short period of time may not be sufficient to trigger mtDNA depletion and subsequent mitochondrial toxicity in this AOP. The maximum lifetime of mitochondria in the kidney cortex has been estimated to be 15 days (Pfeifer and Scheller, 1975). This may explain why effects on KEs downstream of Pol γ inhibition in kidneys of patients and experimental animals generally occur only after continuous exposure for several weeks. This temporal delay between the MIE and the first KE in this AOP is also important to consider when developing *in vitro* test related to KEs in this AOP.

While it is known that mtDNA content and rates of mitochondrial biogenesis differ between tissues, presumably due to tissue-specific energy demands, recent evidence suggests that there are also tissue-specific differences in the mode of mtDNA replication (Herbers et al., 2018). Since the sequence of KEs in this AOP is also relevant to nucleos(t)ide analog toxicity in extrarenal tissues including liver, heart, muscle and the nervous system, tissue-specific rates and modes of mitochondrial biogenesis may be important determinants of the tissue-specific downstream response to a particular nucleos(t)ide analog in addition to toxicokinetic factors as previously discussed (Figure 3).

Moreover, replication of mtDNA and mitochondrial biogenesis are complex processes that are regulated by a range of factors, including nitric oxide (NO), sirtuins, mitogen-activated protein kinase (p38 MAPK), AMP-activated protein kinase (AMPK) and calcium/calmodulin-dependent protein kinase IV (CaMKIV) (Jornayvaz and Shulman, 2010). This suggest that temporal or inter-individual variation in the activity of these pathways may act as modulating factors of the relationship between MIE and KE1. Estrogens are also known to be involved in the control of mitochondrial biogenesis, and thus sex-differences in the KER may exist (Klinge, 2017; Ventura-Clapier et al., 2017).

#### KER2: Depletion of mtDNA Leading to Mitochondrial Dysfunction

It is plausible to assume that the level of depletion of mtDNA required to cause mitochondrial toxicity may be cell- and tissue-specific, with metabolically active cells such as kidney tubule cells being most susceptible. There is a lack of quantitative information on the extent of mtDNA depletion required to induce a significant change in mitochondrial function.

While energy decline is thought to be an immediate consequence of mtDNA depletion, it may further increase mitochondrial stress through generation of oxidative stress that may cause mutations in the mitochondrial genome. It is also possible that an increased mtDNA mutation load due to impaired proofreading activity of Pol γ may contribute to mitochondrial dysfunction. Moreover, nucleos(t)ide analogs often also interact with Pol β, which plays a key role in mtDNA repair and maintenance of mitochondrial genome stability (Prasad et al., 2017). Thus, it needs to be considered that a further pathway initiated by inhibition of DNA Pol β by the very same chemical stressor, leading to increased mtDNA mutations and subsequently altered mitochondrial function, may combine with Pol γ inhibition to cause mitochondrial disturbance (Figure 3).

There are also numerous factors independent of chemical stressors of this AOP (e.g., age, genetic make-up, disease, drug-induced mitochondriopathies, co-exposure) that may affect mitochondrial function and increase the susceptibility of mitochondria to mitotoxicity induced by mtDNA depletion. These modulating factors are depicted in Figure 3.

#### KER3: Mitochondrial Dysfunction Leading to Cytotoxicity

Mitochondrial dysfunction is characterized by a reduced efficiency of oxidative phosphorylation and reduced synthesis of high-energy molecules, such as adenosine-5′-triphosphate (ATP). Expression of toxicity in response to a decline in mitochondrial function may be influenced by the cellular dependence on mitochondrial function, which is known to vary between tissues. Clearly, proximal tubule cells depend on cellular respiration and mitochondrial ATP production to fuel active transport of solutes. However, there is no systematic assessment as to how much decline in mitochondrial function or ATP depletion may be tolerated by a proximal tubule cell before it commits to apoptosis or necrosis. Rather, assays determining mitochondrial activity such as the [3-(4,5-dimethylthiazol-2-yl)-2,5-diphenyltetrazolium bromide] (MTT) assay (succinate dehydrogenase activity) or ATP content (e.g., Cell Titer-Glo^®^) are widely used as cytotoxicity assays based on the assumption that mitochondrial activity is related to the number of viable cells. On the other hand, there are numerous studies employing more than one cytotoxicity assay that show poor correlation between cell viability assays measuring mitochondrial activity vs. other end-points such as compromised plasma membrane integrity (e.g., lactate dehydrogenase (LDH) release), with mitochondrial activity assays generally reported to be more sensitive indicators of cytotoxicity than other endpoints (Fotakis and Timbrell, 2006; Xu et al., 2008). This is consistent with mitochondrial dysfunction being a distinct KE that generally precedes cell demise (Vinken and Blaauboer, 2017). It is important to point out that antivirals as chemical stressors for this AOP may have more than one target and thus several independent pathways may contribute to the overall outcome. Integration into nuclear DNA, telomere shortening, nuclear DNA hypermethylation, interference with ATP synthesis and transport of nucleotides into cellular compartments have all been suggested as possible mechanisms unrelated to mitochondrial dysfunction due to mtDNA Pol γ inhibition (Ho et al., 2000; Moyle, 2000). Biotransformation of cidofovir has been shown to give rise to cidofovir-phosphocholine, which may interfere with synthesis or degradation of membrane phospholipids based on its structural similarity with arabinofuranosyl-cytosine 5′-diphosphocholine (Eisenberg et al., 1998; Ho et al., 2000).

#### KER4: Cytotoxicity (Proximal Tubule Cell) Leading to Kidney Toxicity (Impaired Kidney Function).

Due to the functional reserve of the kidney, homeostasis may be maintained even in the presence of severe kidney damage. It is generally accepted that 70–80% of the renal epithelial mass must be lost before significant changes in serum creatinine (sCr) and blood urea nitrogen (BUN) occur (Pfaller and Gstraunthaler, 1998; Amin et al., 2004). Moreover, even though sCr and BUN are widely used as indicators of renal function in the clinic and in preclinical safety assessment, they are recognized as relatively insensitive markers that only start to rise when renal function is significantly impaired (approximately 50%) (Pfaller and Gstraunthaler, 1998; Amin et al., 2004). Markers related to renal handling of electrolytes, glucose and proteins, including urinary low-molecular weight proteins such as β_2_-microglobulin, cystatin C and neutrophil gelatinase-associated lipocalin (NGAL) may be more sensitive indicators of proximal tubule function, but despite numerous *in vivo* studies investigating nephrotoxic effects of drugs and chemicals, there does not appear to be a systematic quantitative assessment as to the extent of proximal tubule injury required to cause significant changes in these markers. However, a multiscale mathematical model was recently developed and applied to prediction of gentamicin-induced kidney injury based on urinary excretion of kidney injury molecule-1 (Kim-1) (Gebremichael et al., 2018) (see Section 3.5). The authors suggest that the developed model should be generalizable to proximal tubule injury induced by nephrotoxins irrespective of their primary mechanism.

## 3 Receptor-Mediated Endocytosis and Lysosomal Overload Leading to Kidney Toxicity (AOP-257)

**FIGURE 4:**
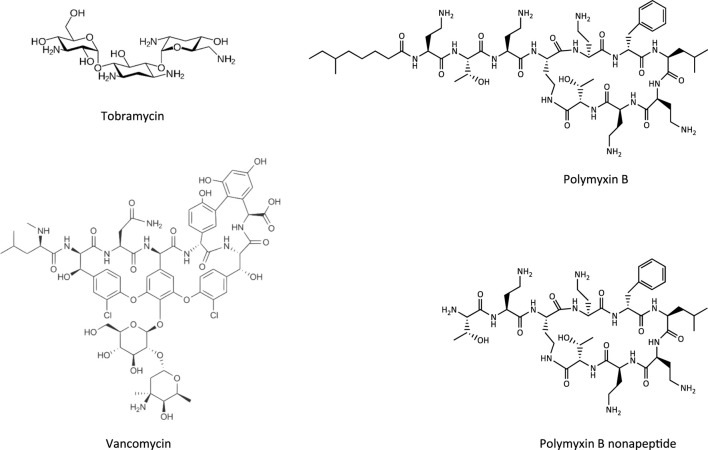
Chemical structures of tobramycin as an example of an aminoglycoside, vancomycin as a glycopeptide, and polymyxin B and its less toxic analogue polymyxin B nonapeptide.

**FIGURE 5:**
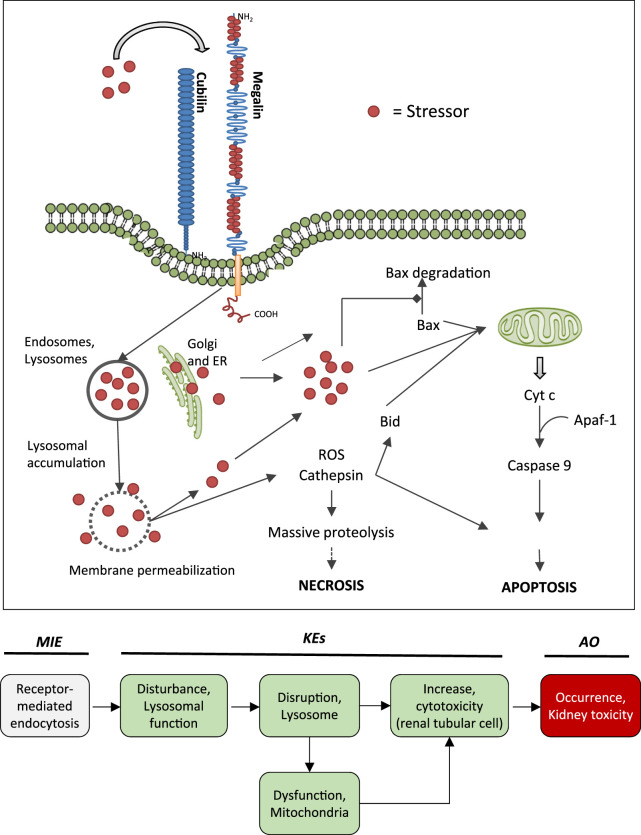
Uptake of polybasic drugs and compounds with peptidic structure via multiligand, endocytic receptors (megalin, cubilin) expressed at the brush-boarder of renal tubule cells. Endocytosed compounds may accumulate within lysosomes, leading to lysosomal swelling and disruption of lysosomes. Release of reactive oxygen species, cytotoxic lysosomal enzymes and possibly also endocytosed compounds may trigger cell death via necrosis or apoptosis. This may occur as a direct consequence (e.g., via massive proteolysis) or secondary to mitochondrial dysfunction. Adverse outcome pathway of receptor mediated endocytosis and lysosomal overload leading to kidney toxicity.

This Adverse Outcome Pathway describes the sequence key events that link receptor mediated endocytosis and lysosomal overload to kidney toxicity. Polybasic drugs and compounds with peptidic structure (e.g., aminoglycosides, glycopeptides, polymyxins) (Figure 4), as well as urinary proteins that act as ligands for multiligand, endocytic receptors (megalin, cubilin) expressed at the brush-boarder of renal tubule cells are efficiently taken up into proximal tubule cell via receptor-mediated endocytosis (Moestrup et al., 1995; Khan and Alden, 2002; Verroust et al., 2002; Thevenod, 2003; Schnellmann, 2013; Liu W. J. et al., 2015). Due to low lysosomal pH, endocytosed compounds may be trapped within lysosomes and accumulate in this organelle, leading to disruption of lysosomal function and eventually permeabilization of lysosomal membranes with release of reactive oxygen species and cytotoxic lysosomal enzymes (Figure 5) (Khan and Alden, 2002; Schnellmann, 2013; Liu D. et al., 2015).

### 3.1 Nephrotoxicity Associated With Lysosomal Accumulation of Ligands of Endocytic Receptors Expressed at the Brush-Boarder of Proximal Tubule Cells

A pivotal function of the renal tubule epithelium is to reabsorb and recycle filtered proteins, carrier-bound vitamins and trace elements from the glomerular ultrafiltrate in order to retrieve nutrients (Eshbach and Weisz, 2017). Cells lining the proximal convoluted tubule are highly specialized for apical endocytosis due to abundant expression of the multiligand endocytic receptors megalin and cubilin at their brush-boarder. Megalin and cubilin ligands include vitamin carrier proteins (e.g., Vitamin D-binding protein, retinol binding protein, transcobalamin), other carrier proteins (e.g., albumin, hemoglobin, liver-type fatty acid-binding protein, metallothionein, transferrin), lipoproteins, enzymes and enzyme inhibitors, immunoglobulin light chains, as well as a number of drugs, including aminoglycosides and polymyxins (Eshbach and Weisz, 2017). Under physiological conditions, endocytosed physiological substrates are efficiently degraded by lysosomal proteases. However, lysosomal swelling and rupture leading to damage of proximal tubule cells may occur if lysosomal degradation is blocked or overwhelmed by substrate overload (Figure 5).

Overproduction of proteins or glomerular injury resulting in increased glomerular permeability and leakage of plasma proteins into urine leads to excessive protein reabsorption and overload of proximal tubule cells. Sustained proteinuria is thus recognized not only as a marker of renal dysfunction but also as a key mediator of tubular injury that contributes to progression of a range of human kidney diseases (Zoja et al., 2004; Erkan et al., 2005). Direct evidence for tubulotoxic effects of excess urinary protein comes from experimental models of albumin or light chain (LC) protein overload nephropathy (Eddy, 1989; Liu et al., 2008; Fang et al., 2018) as well as from *in vitro* studies with isolated kidney tubule cells (Erkan et al., 2001; Li et al., 2008).

Glycopeptide antibiotics, aminoglycosides and polymyxins (Figure 4) are drugs that exhibit bactericidal activity against Gram-positive and/or Gram-negative bacteria. Due to their polybasic structure, these drugs mimic endogenous ligands of endocytic receptors and highjack the endocytic system to enter proximal tubule cells (Avedissian et al., 2019). Polymyxins were first discovered in 1947 as antimicrobial agents with high activity against Gram-negative bacteria, including *Pseudomonas aeruginosa*, *Klebsiella pneumoniae* and *Haemophilus influenzae* (Stansly et al., 1947) based on their ability to disrupt the outer and inner membranes of Gram-negative bacteria after binding to lipopolysaccharide (LPS). Clinical signs of nephrotoxicity such as albuminuria and increased blood non-protein nitrogen were soon after reported in patients administered polymyxin B (Stansly, 1949), limiting their clinical use by the early 1970s. Despite the high risk of nephrotoxicity with conventional doses, polymyxins play an increasingly important role today as salvage therapy of life-threatening multidrug-resistant bacterial infections (Wertheim et al., 2013). The incidence of acute kidney injury following systemic polymyxin therapy with polymyxin B or colistin is reported to range beween 18 and 61% (Justo and Bosso, 2015).

In contrast to polymyxins, the glycopeptide vancomycin and aminoglycosides are mainstream therapy of serious bacterial infections. However, drug-induced kidney injury is a common and dose-limiting adverse effect of vancomycin and aminoglycoside antibiotic treatment. The incidence of acute kidney injury in patients treated with aminoglycoside antibiotics is reported to range between 10–33%, with the wide variation reflecting the characteristics of the population (Lopez-Novoa et al., 2011; McWilliam et al., 2017). The incidence of vancomycin-associated nephrotoxicity is reported to range up to 43%, depending on the target population (Jeffres, 2017).

### 3.2 Mechanism of Proximal Tubule Injury Induced by Aminoglycosides, Polymyxins, Vancomycin and Protein Overload

The key mechanism of kidney injury induced by aminoglycosides is fairly well established (Tulkens, 1989; Lopez-Novoa et al., 2011). Aminoglycosides are rapidly eliminated via urinary excretion (80% within 24 h). Following glomerular filtration, however, 5–10% of the dose are reabsorbed and accumulate within the renal cortex (Antoine et al., 2010). The proximal tubule, and specifically the proximal convoluted tubule, presents the critical target of aminoglycoside nephrotoxicity. The site-specificity of renal toxicity is consistent with the abundant expression of megalin and cubilin, which facilitate efficient uptake into epithelial cells of this nephron segment. Following receptor-mediated endocytosis, aminoglycosides accumulate within the endosomal compartment, particularly within lysosomes (Silverblatt and Kuehn, 1979). Due to their cationic structure, aminoglycosides bind to membrane phospholipids, e.g. within lysosomes, and alter their function (e.g., inhibition of A1, A2, C1 phospholipases). Accumulation of aminoglycosides within lysosomes eventually leads to permeabilization of lysosomal membranes and release of lysosomal content and free aminoglycosides into the cytosol. While cytosolic aminoglycosides have been suggested to directly interfere with the mitochondrial electron transport chain and mitochondrial energy production, and to activate the intrinsic apoptotic pathway, release of cathepsin proteases and reactive oxygen species from lysosomes is on its own detrimental to cells (Lopez-Novoa et al., 2011). Depending on their concentration, cathepsins can induce cell death by either apoptosis through cleavage of caspases and activation of Bid or necrosis through massive proteolysis. Besides lysosomal overload as the major pathway involved in the mechanism aminoglycoside toxicity, it has also been suggested that accumulation of aminoglycosides within the endoplasmic reticulum (ER) may induce ER stress by interfering with protein synthesis and protein folding (Lopez-Novoa et al., 2011).

Following glomerular filtration of polymyxins, renal tubule cells reabsorb 90% of the drug and thus polymyxins accumulate substantially within proximal tubule cells located within the renal cortex (Azad et al., 2019). Although there is some evidence to suggest that polypeptide transporters (PEPT1 and PEPT2) contribute to cellular uptake of polymyxins, megalin-mediated reabsorption is considered to play a key role in accumulation of polymyxins within proximal tubule cells (Yun et al., 2015; Azad et al., 2019). While there is limited data on intracellular distribution of polymyxins in kidney cells (Azad et al., 2019), recent studies in human alveolar epithelial cells demonstrate co-localization of polymyxin B with early endosomes, lysosomes, and mitochondria (Ahmed et al., 2019). Similar to aminoglycosides, polymyxin B was shown to affect release of the hydrolytic lysosomal enzyme *N*-acetyl-β-glucosaminidase from lysosomes *in vitro* (Powell and Reidenberg, 1983). Although there are as yet no mechanistic studies investigating the causal relationship between polymyxin mediated disruption of lysosomal function and death, the evident similarities in renal handling and lysosomal localization suggests that polymyxins act at least in part via the same mechanism as aminoglycosides. Besides lysosomal toxicity, polymyxins have been suggested to cause oxidative stress and apoptosis via mitochondrial, death receptor, and endoplasmic reticulum pathways (Dai et al., 2014; Azad et al., 2019), yet the interlinkage between these effects remains unclear.

Compared to aminoglycosides and polymyxins, the mechanism of vancomycin induced nephrotoxicity is less well studied. However, based on vancomycin being a ligand for megalin and lyososomal accumulation of vancomycin within the S1 and S2 segment of the proximal tubule (Beauchamp et al., 1992; Fujiwara et al., 2012), a similar mechanism can be assumed.

The mechanism of proximal tubule injury induced by protein overload initiated by receptor-mediated endocytosis of urinary proteins induced is also linked to lysosomal dysfunction and membrane permeabilization. Protein overload leads to increased lysosomal number and volume, impaired lysosome-mediated proteolytic degradation as a result of defective lysosomal acidification, and finally lysosomal membrane permeabilization (Liu W. J. et al., 2015). Activation of NF-κB in tubular epithelial cells is thought to play an important role in the progression of tubulointerstitial injury by promoting initerstitial infiltration of mononuclear cells, interstitial edema, and fibrosis (Zoja et al., 2004).

### 3.3 The Adverse Outcome Pathway of Receptor Mediated Endocytosis and Lysosomal Overload Leading to Kidney Toxicity

Binding to multiligand, endocytic receptors expressed at the brush-boarder of renal tubule cells, resulting in proximal tubule cell uptake via receptor-mediated endocytosis can be defined as the molecular initiating event (MIE) in this AOP. Although toxicokinetics are typically not considered as part of an AOP, the molecular interaction between ligand and receptor appears to be essential for the lysosomal accumulation of chemical stressors and subsequent disturbance of lysosomal function (KE1), disruption of lysosomes (KE2) and proximal tubule cell toxicity (KE3) (Figure 5).

Evidence for receptor-mediated endocytosis and lysosomal overload leading to kidney toxicity as an adverse outcome primarily comes from experimental *in vitro* and *in vivo* studies on aminoglycosides, polymyxins, and low molecular weight urinary proteins that serve as chemical stressors for this pathway (Tables 4A–C). In the following sections, evidence supporting the key events and key event relationships in this AOP is presented, followed by a critical assessment of the AOP in terms of temporal and dose-response concordance, essentiality of key events, biological plausibility, coherence, and consistency of the experimental evidence.

**TABLE 4A:** Evidence from human, animal and *in vitro* studies on **aminoglycosides** supporting the key events and qualitative concordance of KEs within this AOP (n/a = not data available).

		Qualitative concordance		
MIE/KE	Short Name	*In vitro*	Animals	Human
MIE	Receptor-mediated endocytosis	• Direct binding of [^3^H]gentamicin to purified gp330 measured by equilibrium dialysis (Moestrup et al., 1995)• Megalin binding of gentamicin demontrated using megalin protein purified from rat renal microvillar membranes; binding inhibited by cilastatin (Hori et al., 2017) • Aminoglycosides (gentamicin, netilimicin, amikacin) inhibit binding of ligand (^125^I-urokinase-PAI) to purified rabbit gp330 (megalin) (Moestrup et al., 1995) • Three-dimensional model describing the complex between megalin and gentamicin (Dagil et al., 2013) • Indirect evidence for binding of gentamicin to gp330 through inhibition of uptake of ^125^I-aprotinin and urokinase PAI-1 complexes in L2 cells and inhibition of binding of ^125^I-aprotinin to rat cortex sections (Moestrup et al., 1995) • Reduced tubular uptake of [^3^H]gentamicin in isolated rat proximal tubules in the presence of a gp330 inhibitor (Moestrup et al., 1995) • Prevention of accumulation and toxicity of aminoglycosides by megalin ligands in opposum kidney cells (Watanabe et al., 2004) • Inhibition of gentamicin accumulation and cytotoxicity proximal tubule derived opossum kidney (OK) cells by statins (Antoine et al., 2010) • Reduced cytotoxicity of gentamicin in the presence of cliastatin (Jado et al., 2014) • Reduced gentamicin uptake in presence of competitive inhibitors (albumin, transferrrin) of receptor mediated endocytosis (Raggi et al., 2011) • Reduced gentamicin uptake and endosomal/lysosomal localization in mouse primary tubule cells obtained from mice models of defective (receptor-mediated) proximal tubule endocytosis (Raggi et al., 2011) • Prevention of neomycin and gentamicin-induced cytotoxicity in LLC-PK1 cells by the megalin ligand apolipoprotein E3 (Takamoto et al., 2005a; Takamoto et al., 2005b)	• Reduced nephrotoxicity of gentamicin (80 mg/kg bw, i.p, 9 days) in rats in the presence of cliastatin (Jado et al., 2014) • Prevention of renal accumulation and toxicity of aminoglycosides by megalin ligands in mouse kidney (Watanabe et al., 2004) • Functional or genetic megalin deficiency affords protection from renal accumulation of gentamicin in mice (Schmitz et al., 2002) • Reduced renal aminoglycoside accumulation, intra-lysosomal localization and proximal tubule dysfunction (proteinuria) in mice models of defective (receptor-mediated) proximal tubule endocytosis (Raggi et al., 2011) • Subcellular localization of [^3^H]gentamicin in lysosomes of proximal tubule cells of rats and mice administered [^3^H]gentamicin (Schmitz et al., 2002) • Subcellular localization of tobramycin in lysosomes of proximal tubule cells of rats treated with tobramycin for 10 days (Beauchamp et al., 1992)	• Localization of gentamicin in variably sized granular structures in the cytoplasm of proximal tubular cells indictive of intralysosomal accumulation in renal biopsies obtained from patients administered gentamicin (De Broe et al., 1984)
KE1	Disturbance, Lysosomal function	• Induction of lysosomal phospholipidosis in cultured rat fibroblasts by gentamicin associated with decreased lysosomal sphingomyelinase activity (Aubert-Tulkens et al., 1979; Tulkens and Van Hoof, 1980) • Inhibition of phosphatidylinositol phospholipase C from rat renal tissue by aminoglycosides (Lipsky and Lietman, 1982) • Inhibition of lysosomal enzyme release from isolated renal cortical lysosomes by low concentrations of aminoglycosides (Powell and Reidenberg, 1982; 1983)	• Ultrastructural changes in renal proximal tubule “cytosomes”, including increased lysosomal size and formation of myeloid bodies in F344 rats s.c. treated with tobramycin (40 and 120 mg/kg bw) or gentamicin (40 mg/kg bw) for up to 14 days (Houghton et al., 1978b). • Increase in number and size of secondary lysosomes with myeloid bodies within lysosomes and increased phospholipid conent of renal cortex in rats injected with gentamicin (100 mg/kg bw, 4 days) (Kaloyanides and Pastoriza-Munoz, 1980) • Phospholipidosis (evidenced by accumulation of phosphatidylinositol) indicative of impaired lysosomal degradation through inhibition of lysosomal phospholipases in renal cortex of rats injected with gentamicin or netilmicin (100 mg/kg bw, 1–2 days) (Feldman et al., 1982) • Myeloid bodies in urinary sediment of rats treated with aminoglycosides (Mandal and Bennett, 1988)	• Myeloid bodies in proximal tubules in renal biopsies and nephrectomies obtained from patients receiving gentamicin (Houghton et al., 1978a) • Lysosomal alterations (e.g. increased volume and density, decreased lysosomal phospholipase A activity, containing dense lamellar and concentric structures = phospholipids organized in bilayers) in kidney biopsies obtained from patients treated with aminoglycosides (De Broe et al., 1984; Roels et al., 1984) • Intralysosomal myeloid bodies in urinary sediment of patients treated with aminoglycosides (Mandal et al., 1987)
KE2	Disruption, Lysosome	• Enhanced release of lysosomal enzymes from isolated renal cortical lysosomes at high concentrations of aminoglycosides (Powell and Reidenberg, 1983) • Lysosomal membrane permeabilization in gentamicin-treated renal LLC-PK1 cells (Servais et al., 2005; Denamur et al., 2011)	• Ruptured “cytosomes” in proximal tubule cells of F344 rats s.c. treated with tobramycin (40 and 120 mg/kg bw) or gentamicin (40 mg/kg bw) for up to 14 days (Houghton et al., 1978b)	• Myeloid bodies in urinary sediment of patients treated with aminoglycosides (Mandal et al., 1987)
KE3	Increase, Cytotoxicity	• Concentration-dependent increase in apoptosis in porcine proximal tubule cells (Jado et al., 2014) and LLC-PK1 cells (Servais et al., 2005; Denamur et al., 2011) treated with gentamicin	• Vacuolization and hyaline casts in the tubular lumen of rats treated with gentamicin (80 mg/kg bw, i.p., 9 days) (Jado et al., 2014) • Proximal tubule necrosis in F344 rats treated with tobramycin or gentamicin for up to 14 days (Houghton et al., 1978b) • Inhibition of proximal tubular transport processes by aminoglycosides, and proximal tubular cell necrosis (Kaloyanides and Pastoriza-Munoz, 1980)	• Cellular casts and shedded tubular cells with embedded myeloid bodies in urinary sediment of patients treated with aminoglycosides (Mandal et al., 1987) • Increased N-acetyl-beta-d-glucosaminidase (NAG) activity in urine of patients treated with aminoglycosides indicative of proximal tubule injury (Wiland and Szechcinski, 2003) (Glass et al., 2005; Etherington et al., 2007)
AO	Occurrence, Kidney Toxicity	–	• Increased sCrea, BUN and proteinuria in rats treated with gentamicin (80 mg/kg bw, i.p., 9 days) (Jado et al., 2014) • Increased sCrea and BUN in F344 rats s.c. treated with tobramycin (40 and 120 mg/kg bw) or gentamicin (40 mg/kg bw) for up to 14 days (Houghton et al., 1978b) • Incrased BUN and reduced creatinine clearance in F344 rats s.c. treated with gentamicin or tobramycin (40 mg/kg bw) for up to 10 days (Mandal and Bennett, 1988)	• Nephrotoxicity and acute kidney injury in patients who receive aminoglycoside therapy (Bertino et al., 1993; Oliveira et al., 2009; Lopez-Novoa et al., 2011; Zappitelli et al., 2011) • Reduced glomerular filtration rate, increased BUN and sCrea, oligo-anuric renal failure in patients treated with aminoglycosides (Wiland and Szechcinski, 2003)

**TABLE 4B:** Evidence from human, animal and *in vitro* studies on **polymyxins** supporting the key events and qualitative concordance of KEs within this AOP (n/a = not data available).

		Qualitative concordance		
MIE/KE	Short Name	*In vitro*	Animals	Human
KE1	Disturbance, Lysosomal function	n/a	n/a	n/a
				
KE2	Disruption, Lysosome	• Polymyxin-mediated release of N-acetyl-β-(d)-glucosaminidase from kidney lysosomes (Powell and Reidenberg, 1983)	• Increased urinary excretion of N-acetyl-β-(d)-glucosaminidase in C57BL/6J mice (Hori et al., 2017) and rats treat treated with colistin (1 mg/kg bw; i.v.) (Suzuki et al., 2013)	• Increased N-acetyl-β-d-glucosaminidase (NAG) activity in urine of patients treated with colistin indicative of proximal tubule injury (Etherington et al., 2007)
				
KE3	Increase, Cytotoxicity	n/a	• Proximal tubule injury evidenced by histopathology and kidney injury molecule-1 (KIM-1) expression exclusively in megalin-replete proximal tubule cells of kidney-specific mosaic megalin knockout mice treated with colistin (Hori et al., 2017) • Increased urinary excretion of N-acetyl-β-(d)-glucosaminidase, tubular vacuolization, tubular dilation or atrophy, brush border loss, tubular cell lysis, and cast formation; and increased KIM-1 expression in C57BL/6J mice treated with colistin (Hori et al., 2017)	n/a
				
AO	Occurrence, Kidney Toxicity	-	n/a	• Acute kidney injury in patients treated with polymyxins (Kubin et al., 2012; Alvarado Reyes et al., 2019)

**TABLE 4C:** Evidence from human, animal and *in vitro* studies on **vancomycin** supporting the key events and qualitative concordance of KEs within this AOP (n/a = not data available).

		Qualitative concordance		
MIE/KE	Short Name	*In vitro*	Animals	Human
KE1	Disturbance, Lysosomal function	n/a	• Increased numbers of lysosomes in renal tubule cells of rabbits treated with vancomycin as evidenced by electron microscopy (Toyoguchi et al., 1997)	n/a
				
KE2	Disruption, Lysosome	n/a	• Increased urinary excretion of N-acetyl-β-d-glucosaminidase in rabbits treated with vancomycin (Toyoguchi et al., 1997)	n/a
				
KE3	Increase, Cytotoxicity	• Concentration dependent increase in apoptosis in porcine renal proximal tubular epithelial cells treated with vancomycin (Humanes et al., 2015)	• Proximal tubule injury evidenced by histopathology and kidney injury molecule-1 (KIM-1) expression exclusively in megalin-replete proximal tubule cells of kidney-specific mosaic megalin knockout mice treated with vancomycin, (Hori et al., 2017) • Tubular dilatation, destruction of renal tubule epithelial cells, hyaline casts in rabbits treated with vancomycin (Toyoguchi et al., 1997) • Proximal tubule injury evidenced by histopathology and increased urinary kidney injury molecule-1 (KIM-1) in rats treated with vancomycin (O'Donnell et al., 2017) • Acute tubular necrosis in mice dosed with vancomycin (Luque et al., 2017)	• Biopsy proven acute tubule necrosis in patients treated with vancomycin (Sokol et al., 2004; Wicklow et al., 2006; Wu et al., 2007; Shah-Khan et al., 2011; Belen et al., 2012; Htike et al., 2012; Tantranont et al., 2019) (Luque et al., 2017)
				
AO	Occurrence, Kidney Toxicity	–	• Incrased sCrea and BUN and increased kidney to body weight in rabbits treated with vancomycin (Toyoguchi et al., 1997) • Acute renal failure in mice dosed with vancomycin (Luque et al., 2017)	• Acute kidney injury in patients receiving vancomycin therapy (Elyasi et al., 2012; Sinha Ray et al., 2016)

#### Molecular Initiating Event: Receptor-Mediated Endocytosis

Receptor-mediated endocytosis via binding to the multi-ligand receptor system megalin/cubilin constitutes the principle pathway of cellular uptake of polybasic drugs and low molecular weight proteins (LMWP) from the glomerular filtrate. The interaction of polybasic drugs and LMWPs with the receptor located at the brush border is facilitated by the negative charge of acidic membrane phospholipids and interaction of basic residues of the ligand with negatively charged receptor domains (Moestrup et al., 1995). The site-specific toxicity of polybasic drugs and LMWPs to the proximal tubule, the proximal convoluted tubule, corresponds with the abundant expression of megalin and high endocytic capacity within this nephron segment (Schuh et al., 2018). Receptor-mediated endocytosis is primarily responsible for delivery of polybasic drugs and LMWPs to lysosomes and subsequent disturbance of lysosomal function that ultimately leads to kidney toxicity. There are several lines of *in vitro* and *in vivo* evidence that support receptor-mediated endocytosis as the key initiating event: 1) stressors for this AOP are ligands of megalin, 2) uptake and subsequent toxicity of stressors for this AOP can be prevented by competitive inhibitors and indirectly by statins, which block post-translational prenylation of guanosine-5′-triphosphate (GTP)-binding proteins are required for megalin-mediated endocytosis (Antoine et al., 2010; McWilliam et al., 2018). 3) Loss of megalin, e.g., through megalin knockout, protects from accumulation and toxicity of stressors. Experimental evidence from *in vitro* and *in vivo* studies on aminoglycosides, polymyxins and vancomycin as chemical stressors of this AOP are summarized in Tables 4A–C. In addition to these chemical stressors, silencing of megalin and cubulin has been shown to inhibit myeloma light chain endocytosis and reduce the toxicity of myeloma light chains (Li et al., 2008), which are exessively produced in multiple myeloma and may cause proximal tubule alterations through overload of the endocytic process (Batuman, 2007). Similarly, siRNA mediated knockdown of megalin and cubilin was shown to block albumin-induced tubular injury (Liu D. et al., 2015). Although there are as yet no data related to the MIE in humans, it is noted that a phase IIa randomized controlled clinical trial investigating prevention of aminoglycoside-induced kidney injury by rosuvastatin in children with cystic fibrosis has been initiated (McWilliam et al., 2018).

#### Key Event 1: Disturbance, Lysosomal Function

There is substantial evidence from *in vitro* and *in vivo* studies that accumulation of endocytosed drugs and LMWPs within lysosomes leads to ultrastructural changes, including increased number and size of lysosomes in renal proximal convoluted cells (Houghton et al., 1978b; Kaloyanides and Pastoriza-Munoz, 1980; Feldman et al., 1982; Mandal and Bennett, 1988; Toyoguchi et al., 1997). These ultastructural changes are considered to occur as a consequence of substrate overload or reduced lysosomal proteolytic capacity due to compound binding to lysosomal phospholipid membrane and inhibition of phospholipases. Interference with phospholipid metabolism results in accumulation of phospholipids with formation of lysosomal myeloid bodies, i.e. concentric multilaminar phospholipid membrane whorls. Experimental *in vitro* and *in vivo* studies demonstrating inhibition of phospholipases, phospholipidosis and myeloid body formation by chemical stressors for this AOP as well as detection of myeloid bodies in renal biopsies and urinary sediment of patients treated with aminoglycosides support disturbed lysosomal function as a key event in this AOP (Tables 4A–C). In addition to interference with lysosomal phospholipid metabolism, lysosomal protein overload through excessive exposure of proximal tubule cells to ligands of the endocytic receptor may also lead to altered lysosomal function that expresses itself in hyaline droplet formation. Recent data suggest that changes in the tertiary structure of albumin may interfere with lysosomal proteolysis (Medina-Navarro et al., 2019). This is reminiscent of stabilization of α2u-globulin through binding of chemicals to α2u-globulin, resulting in resistance of the α2u-globulin–chemical complexes to lysosomal degradation and subsequent protein droplet formation in α2u-nephropathy (Lehman-McKeeman et al., 1990).

#### Key Event 2: Disruption, Lysosome

While reduced release of N-acetyl-β-d-glucosaminidase suggestive of lysosomal membrane stabilization may be an initial response to chemical stressors of this AOP (Powell and Reidenberg, 1982; Powell and Reidenberg, 1983), swelling of lysosomes due to intralysosomal accumulation of chemical stressors and macromolecules (protein, phospholipids) ultimately leads to lysosomal membrane permeabilization or lysosome rupture. It has been suggested that impaired phospholipid metabolism may increase the hydrophobicity of the lysosomal membrane, thereby interfering with transport of water-soluble products across the lysosomal membrane and subsequent osmotic disruption of lysosomes (Powell and Reidenberg, 1982; Powell and Reidenberg, 1983). In addition to the evidence for chemical stressors presented in Tables 4A–C, proximal tubule toxicity induced by albumin and urinary proteins has been shown to involve lysosomal membrane permeabilization and lysosome rupture (Liu D. et al., 2015; Liu W. J. et al., 2015). As a result of lysosomal disruption, lysosomal enzymes such as cathepsins are released into the cytosol (Lopez-Novoa et al., 2011), which may be evident by reduced lysosomal cathepsin activity or immunoreactivity (Li et al., 2000).

#### Key Event 3: Increase, Cytotoxicity (Renal Tubular Cell)

The link between disruption of lysosomes and cell death is well established (Bursch, 2001; Turk et al., 2002; Guicciardi et al., 2004). Leakage of lysosomal proteases such as cathepsins may trigger apoptosis directly through activation of pro-caspases or indirectly via promoting release of cytochrome C from mitochondria, whereas extensive lysosomal rupture results in necrosis. Evidence for this comes from studies demonstrating that controlled lysosomal rupture induced by a lysosomoropic detergent causes cathepsin release prior to apoptosis (Li et al., 2000). This study also shows that changes in mitochondrial membrane potential occur seondary to lysomal rupture (Li et al., 2000). There is ample evidence from *in vitro* experiments and studies in animals and humans that demonstrate proximal tubule toxicity of stressors of this AOP (Tables 4A–C). On a cautionary note, establishment of stable cell lines often involves use of aminoglycosides as selection antibiotics and thus renal cell lines generated via this protocol may be resistant to aminoglycoside toxicity.

#### Adverse Outcome: Kidney Toxicity

The link between proximal tubule injury and impaired kidney function has already been described in Section 2.3. Since receptor-mediated endocytosis occurs primarily within the S1 segment of the proximal tubule, which is also the primary site of glucose reabsorption, increased urinary glucose is often one of the earliest signs of proximal tubule injury induced by stressors of this AOP. Increased urinary excretion of (low-molecular-weight) proteins that are normally endocytosed and degraded is also frequently observed as an early response to stressors of this AOP, although it is not entirely clear if such changes necessarily always reflect impaired tubular reabsorption as a result of tubule damage or rather competitive inhibition of receptor-mediated endocytosis by the chemical stressor of this AOP. With increasing severity of tubule damage, nephrotoxicity induced by stressors of this AOP may progress to changes in blood urea nitrogen (BUN) and serum creatinine (sCrea), reduced glomerular filtration, and oligo-anuric renal failure. Such changes are evident in experimental animals treated with aminoglycosides and polymyxins as well as in patients receiving aminoglycoside, glycopeptide and polymyxin antibiotics (Tables 4A–C).

### 3.4 Assessment of the Adverse Outcome Pathway of Receptor Mediated Endocytosis and Lysosomal Overload Leading to Kidney Toxicity

#### Biological Plausibility

The mechanistic basis for a causal relationship between the KEs in this AOP is detailed in Section 3.3. Considering the high endocytic activity of convoluted proximal tubule cells, the physiological role of lysosomes in the degradation of endocytosed material, the proteolytic function of lysosomal enzymes and toxicity of highly reactive oxygen species that leak into the cytosol upon lysosomal membrane permeabilization subsequent to lysosomal overload, and the critical role of the proximal tubule for kidney function, the level of confidence in the biological plausibility of key event relationships (KERs) within the proposed AOP can be considered as high (Table 5).

**TABLE 5:** Weight-of-evidence analysis of KERs in the adverse outcome pathway of receptor-mediated endocytosis and lysosomal overload leading to kidney toxicity.

KE Upstream	KE Downstream	Weight of evidence (WoE) for KER			
		Biological Plausibility	Essentiality	Empirical support	Overall WoE
Receptor-mediated endocytosis	Disturbance, Lysosomal function	high	high	moderate	high
Disturbance, Lysosomal function	Disruption, Lysosome	high	moderate	moderate	high
Disruption, Lysosome	Increase, Cytotoxicity	high	high	high	high
Increase, Cytotoxicity	Occurrence, Kidney Toxicity	high	high	high	high

#### Essentiality of Key Events

Essentiality of the MIE in this AOP is clearly supported by a range of *in vitro* and *in vivo* studies in experimental animals that demonstrate reduced cellular uptake, accumulation and cytotoxicity of model stressors for this pathway in megalin deficient kidney cells or in the presence of competitive inhibitors of receptor mediated endocytosis, e.g., (Moestrup et al., 1995; Schmitz et al., 2002; Watanabe et al., 2004; Takamoto et al., 2005a; Takamoto et al., 2005b; Wolff et al., 2006; Wolff et al., 2008; Raggi et al., 2011; Onodera et al., 2012; Suzuki et al., 2013; Liu W. J. et al., 2015; Hori et al., 2017) (Tables 4A–D) and can thus be considered high (Table 5). Pharmacological inhibition of cathepsins has been shown to ameliorate protein overload-triggered tubule cell apoptosis (Liu W. J. et al., 2015), providing evidence that lysosomal membrane permeabilization and associated cathepsin release is an essential trigger for cell death in this pathway. Similarly, Song et al. (2017) showed that caspase-3 activation and apoptosis caused by lead-induced lysosomal membrane permeabilization in primary rat proximal tubular cells is significantly reduced by cathepsin B and D inhibitors (Song et al., 2017). Based on direct evidence for essentiality of the MIE and an important KE upstream of cytotoxicity, the level of confidence for essentiality of KEs in this AOP can thus be considered as high (Table 5).

#### Empirical Evidence: Dose-Response and Temporal Concordance

There are numerous studies that provide dose-response data on aminoglycoside nephrotoxicity in experimental animals through comparative analysis of histopathological changes, clinical chemistry parameters indicative of renal function, and novel biomarkers of kidney injury. These studies frequently report proximal tubule injury (KE3) at doses lower than those required to induce a significant decline in kidney function (Table 6). In contrast, there are only few studies that considered early upstream KEs in this AOP, i.e. lysosomal alterations (Table 6). While it is not possible from the available data to conclude that aminoglycoside-mediated effects on lysosomes occur at lower doses compared to those required to induce proximal tubule injury and kidney failure, it is evident that these lysosomal changes are recorded at an equal dose.

**TABLE 6:** Dose-Time Concordance of KEs based on rodent studies with gentamicin as a specific stressor for the adverse outcome pathway of receptor-mediated endocytosis and lysosomal overload leading to kidney toxicity (n.d. = not determined; n/a = not data available)).

			Temporal concordance				
	Species	Dose (mg/kg bw)	1 day	3 days	6–7 days	10–14 days	References
**Dose-response concordance**	Rats	25	KE1 n.d KE2 n.d KE3 PT injury AO -	KE1 n.d KE2 n.d KE3 - AO -	n/a	KE1 - KE2 - KE3 PT injury AO -	Hoffmann et al. (2010)

		40	KE1 n.d KE2 n.d KE3 PT injury AO -	KE1 myeloid bodies KE2 n.d KE3 - AO kidney function ↓	KE1 myeloid bodies KE2 “cytosome” rupture KE3 PT injury AO kidney function ↓	n/a	Houghton et al. (1978b)

		60	KE1 n.d KE2 n.d KE3 PT injury AO -	KE1 n.d KE2 n.d KE3 PT injury AO -	KE1 n.d KE2 n.d KE3 PT injury AO -	n/a	Sieber et al. (2009)

		75	KE1 n.d KE2 n.d KE3 PT injury AO -	KE1 n.d KE2 n.d KE3 PT injury AO -	n/a	KE1 n.d KE2 n.d KE3 PT injury AO kidney function ↓	Hoffmann et al. (2010)

		100	KE1 phospholipidosis KE2 n.d KE3 n.d AO n.d	n/a	n/a	n/a	Feldman et al. (1982)

		120	KE1 n.d KE2 n.d KE3 PT injury AO -	KE1 n.d KE2 n.d KE3 PT injury AO kidney function ↓	KE1 n.d KE2 n.d KE3 PT injury AO kidney function ↓	n/a	Sieber et al. (2009)

PT injury as evidenced by histopathology or change in biomarker indicative of proximal tubule injury or dysfunction. Decline in kidney function as evidenced by a significant change in serum creatinine or blood urea nitrogen.

Collectively, the available *in vitro* and *in vivo* studies conducted using chemical stressors for this AOP generally demonstrate effects on KEs across the entire AOP at equal doses/concentrations of the stressor, with some evidence for upstream events occurring at lower concentrations than downstream KEs. There are no data that would disagree with the assumption of dose-response concordance. Based on the criteria for assessing AOP (Box 1), the level of confidence for concordance of dose-response can thus be regarded as high.

There are some studies investigating the time-course of aminoglycoside nephrotoxicity in rats that collectively support the temporal sequence of KEs in this AOP (Table 6). While many of these studies focused on analysing the time-course of aminoglycoside-induced histopathological changes and impact on kidney function, there are also some studies that demonstrate that injury to the convoluted proximal tubule occurs subsequent to lysosomal changes. In Fischer F344 rats treated with either gentamicin or tobramycin, ultrastructural changes, most prominently vacuolar structures containing myeloid bodies (referred to as cytosomes by the authors) were recorded in proximal tubules prior to any other evidence of injury (Houghton et al., 1978b). In this study, increased numbers of “cytosomes” and “cytosomal” rupture were observed concomitant with tubule cell injury (Houghton et al., 1978b). In a further study in rats, phospholipidosis was detected in the renal cortex within 24 h of a single dose of gentamicin or netilmicin, leading the authors to conclude that alterations in phosholipid metabolism are an early event in the pathogenesis of aminoglycoside toxicity that precedes signs of tubule injury (Feldman et al., 1982). *In vitro*, lysosomal membrane permeabilization in gentamicin-treated renal LLC-PK_1_ cells was shown to precede mitochondrial changes and apoptosis (Servais et al., 2005; Denamur et al., 2011). Similarly, recent time-resolved analyses conducted within the frame of the Risk-IT project showed that cytotoxicity induced by polymyxin B was preceded by a decrease in lysosomal number, thus supporting the temporal sequence of events within this AOP (Jarzina et al., 2022).

Further support for the sequence of KEs comes from studies in patients receiving aminoglycoside therapy. A retrospective analysis of renal biopsies obtained from patients that received gentamicin within 6 weeks of biopsy reported ultrastructural changes in renal proximal tubule lysosomes in the absence of clinical signs of nephrotoxicity (Houghton et al., 1978a). Similarly, early lysosomal changes were observed in proximal tubular cells of patients receiving therapeutic doses of aminoglycosides for four consecutive days prior to nephrectomy (De Broe et al., 1984). Although no additional histopathological or clinical chemistry data were reported, the authors suggested that these lysosomal alterations occur before the onset of excretory failure (De Broe et al., 1984). In urine samples obtained from 20 patients receiving aminoglycoside therapy for 3–26 days, myeloid bodies were found in urinary sediment irrespective of whether or not the patient developed acute renal failure, although the number of myeloid bodies was increased in patients with acute renal failure (Mandal et al., 1987). In contrast, both the incidence of the appearance of renal tubule cells in the urinary sediment and their number was significantly increased in patients with aminoglycoside-induced renal failure as compared to the non-renal failure group, thus supporting the temporal sequence of events leading from lysosomal alterations to proximal tubule dysfunction and necrosis and ultimately renal excretory failure (Mandal et al., 1987).

Increased activity of the lysosomal enzyme N-acetyl-β-d-glucosaminidase (NAG) was observed in urine of patients treated with tobramycin or colistin in the absence of changes in sCrea and BUN (Etherington et al., 2007).

#### Weight-Of-Evidence Analysis

Based on biological plausibility and empirical support, the overall weight-of-evidence of KERs in this AOP can be considered as high (Table 5).

### 3.5 Quantitative and Temporal Understanding of Key Event Relationships

Based on the available literature, there is at present little or no quantitative information on the response-response relationship between two pairs of KEs in this AOP.

#### KER1: Receptor-Mediated Endocytosis Leading to Disturbance of Lysosomal Function

Numerous studies demonstrate that inhibition of ligand binding and receptor-mediated endocytosis reduces toxicity of the chemical stressor. However, there are no data to describe the quantitative relationship between receptor-mediated endocytosis and disturbance of lysosomal function. While being an essential and thus indispensable component in this AOP, the MIE receptor-mediated endocytosis directly links toxicokinetics to molecular and cellular responses. As such, the relationship between the MIE and KE1 may no longer be chemical-agnostic when moving from qualitative descriptions to quantitiative AOPs. Rather, determination of intralysosomal or intracellular accumulation of the stressor may present the best quantifiable measure of receptor-mediated endocytosis, whereby the intracellular stressor concentration necessary to impair lysosomal function may vary between stressors. Not suprisingly, within a group of structurally related compounds such as polymyxin antibiotics, there is a positive correlation between biological responses and affinity to endocytic receptors at the brush boarder membrane or renal accumulation (Vaara et al., 2008; Keirstead et al., 2014; Jarzina et al., 2022).

#### KER2: Disturbance of Lysosomal Function Leading to Disruption of Lysosomes

There is as yet little information as to the degree of disturbance of lysosomal function necessary to cause permeabilization of lysosomal membranes and release of lysosomal content. Within the Risk-IT project, the response-response relationship betweeen lyososmal membrane associated proteins (LAMP-1/2), reflecting disturbed lysosomal function (KE1), and release of cathepsin D from lysosomes as an endpoint reflecting lysosomal disruption (KE2) was established from experimental data on polymyxin B and successfully employed to predict the downstreamt KE of structural analogs based on experimental KE1 data (Jarzina et al., 2022).

#### KER3: Disruption of Lysosomes Leading to Increased Proximal Tubule Cytotoxicity

While a response-response relationship between lysosomal permeabilization and proximal tubule cytotoxicity has not yet been established, a study investigating controlled lysosomal rupture by the synthetic lysosomotropic detergent O-methyl-serine dodecylamide hydrochloride (MSDH) in a murine macrophage cell line provides important quantitative and temporal information on this KER (Li et al., 2000). At low concentrations of the lysosomotropic detergent, lysosomal membrane destabilization was observed by reduced acridine orange fluoresence intensity and granularity scoring of cathepsin D immunoreactivity. Lysosomal leakage preceded morphological signs of apoptosis, activation of caspase-3-like proteases and mitochondrial changes, indicating that cell death occurred secondary to partial lysosomal rupture, presumably due to the apoptotic role of lysosomal proteases (Li et al., 2000; Turk et al., 2002). In contrast, extensive lysosomal rupture, e.g., induced by high concentrations of MSDH, results in necrosis as the predominant type of cell death (Li et al., 2000; Turk et al., 2002). In an attempt to define the quantitative relationship between disruption of lysosomes and proximal tubule cytotoxicity, the response-response between cathepsin D release from lysosomes and cytotoxicity was established from experimental data on polymyxin B. Although there was some concern regarding the reliability of the *in vitro* cathepsin assay, the response-response relationship was successfully employed for prediction of cytotoxicity of structural analogs (Jarzina et al., 2022).

#### KER4: Proximal Tubule Cytotoxicity Leading to Kidney Toxicity

Considering that proximal tubule epithelial cell injury is a common key event involved in various AOPs that lead to acute and/or chronic kidney injury, Gebremichael et al. (2018) developed a multiscale quantitative systems pharmacology model to relate drug induced proximal tubule cell injury (i.e., a celluar event) to renal dysfuction (i.e., an adverse outcome at organ level) (Gebremichael et al., 2018). The model is based on the assumption that “the relationships between cell injury and death and subsequent effects on tubular dysfunction, biomarker expression, and organ-level dysfunction should be independent of the injury mechanism” (Gebremichael et al., 2018) and thus independent of the nephrotoxic agent (i.e., chemical agnostic). Thus, the authors consider that their mathematical model should be applicable to prediction of drug-induced changes in kidney function based on the extent of cell injury and cell death inferred from urinary biomarker responses. The model, which consists of a cellular injury submodel and a systems renal physiology model, was developed using histopathology and biomarker data obtained from a single dose cisplatin study in rats. Model parameters were fitted to the experimentally established time-course of urinary biomarker responses (Kim-1, albumin, glucose, αGST) to determine the fractions of functional, injured and dead proximal tubule cells, and subsequently to simulate serum creatinine levels as a readout for alterations in kidney function. The model was successfully applied not only to predict the serum creatinine time course in response to repeated cisplatin administration but also in response to gentamicin as a structurally unrelated drug based on urinary Kim-1 data. Thus, the developed model holds great promise for translation of time-resolved urinary biomarker data as proxy of proximal tubule cell injury and death into the time-course and severity of proximal tubule injury and organ-level dysfunction. Moreover, combined with quantitative *in vitro* to *in vivo* extrapolation, the model may help to bridge the gap between *in vitro* and *in vivo* responses by facilitating prediction of kidney injury and dysfunction based on *in vitro* cytotoxicity data obtained in proximal tubule cells.

## 4 Renal Protein Alkylation Leading to Kidney Toxicity (AOP-258)

**FIGURE 6:**
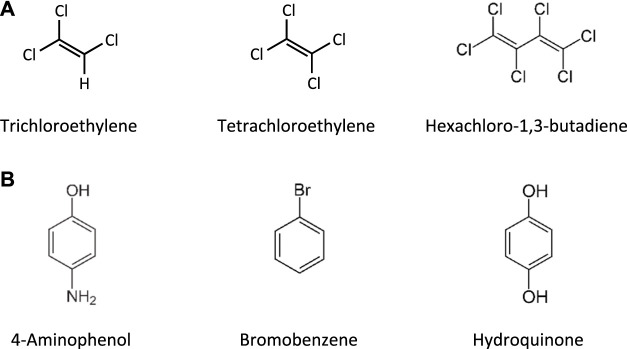
Chemical structures of nephrotoxic haloalkenes **(A)** and phenols and heteroatom-substituted benzene derivatives that are bioactivated to nephrotoxic quinones **(B)**.

**FIGURE 7:**
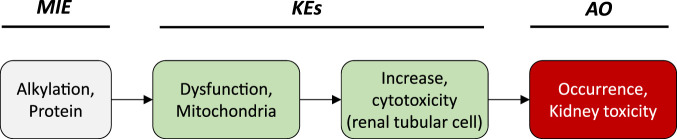
Adverse outcome pathway of renal protein alkylation leading to kidney toxicity.

This Adverse Outcome Pathway describes the sequential key events that link protein alkylation to kidney toxicity. It is well established that bioactivation of xenobiotics to reactive intermediates that covalently bind to proteins presents a major mechanism by which xenobiotics may cause proximal tubule injury. Examples for compounds that form covalent protein adducts in proximal tubule cells include haloalkenes (e.g., trichloroethylene, tetrachloroethylene, hexachloro-1,3-butadiene, chloroform), quinones (derived from e.g. hydroquinone, bromobenzene, 4-aminophenol), cephalosporins, and N-(3,5-dichlorophenyl)succinimide (Birner et al., 1994; Lau, 1995; Tune, 1997; Griffin and Harvison, 1998; Kleiner et al., 1998; Pahler et al., 1998) (Figure 6). Covalent interaction of a chemical or a metabolite with cellular proteins represents the molecular initiating event (MIE) that triggers perturbation of cellular functions, of which mitochondrial dysfunction leading to ATP depletion appears to be most critical for proximal tubule cell death by apoptosis and/or necrosis (Figure 7) (Aleo et al., 1991; Groves et al., 1991; Hill et al., 1992; Tune, 1997; Chen et al., 2001). Alternative events that may contribute to toxicity include endoplasmic reticulum (ER) stress, glutathione depletion and oxidative stress (van de Water et al., 1996). Tubular obstruction and inflammatory responses to proximal tubule injury including activation of complement may cause secondary toxicity and thus amplify kidney injury, resulting in a progressive decline in kidney function (evidenced by e.g. rise in sCrea and BUN).

### 4.1 Nephrotoxicity and Mechanism of Kidney Injury Induced by Agents That Covalently Bind to Proteins

Ever since the pioneering work of the Millers on the role of liver protein alkylation in the carcinogenicity of aminoazo dyes (Miller and Miller, 1947; Miller and Miller, 1966) and subsequent work by Brodie and co-workers demonstrating that covalent protein binding of acetaminophen precedes hepatic necrosis (Jollow et al., 1973), covalent modification of critical target proteins has been established as an important mechanism of toxicity mediated by reactive intermediates. While intial work in this field focussed on liver toxicity and/or carcinogenicity, the concept of protein alkylation leading to toxicity is universal and not restricted to a particular target tissue. Rather, it is often the site of bioactivation to a chemically reactive intermediate that determines the site of toxicity. Selective nephrotoxicity of several haloalkenes, such as hexachlorobutadiene (HCB), trichloroethene (TRI) and perchloroethene (PER), and hydroquinones and aminophenols frequently involves formation of glutathione-*S*-conjugates (Dekant, 2001). Although conjugation with glutathione (GSH) occurs largely in the liver, it serves to direct xenobiotics to the kidneys. By the sequential action of γ-glutamyltransferase (GGT) and aminopeptidases/dipeptidases that are abundantly expressed by renal tubule cells, glutathione *S*-conjugates delivered to the kidneys are cleaved to the corresponding cysteine *S*-conjugates. Following active uptake into kidney epithelial cells via organic-anion transporters, the cysteine *S*-conjugates of haloalkenes may undergo cysteine conjugate β-lyase-mediated cleavage to a reactive intermediate capable of covalent binding to cellular proteins. In contrast, the nephrotoxicity of aminophenol and hydroquinone *S*-conjugates does not depend on β-lyases but appears to be linked to oxidation to a reactive quinone (Dekant, 2001).

Hexachloro-1,3-butadiene (HCBD) is a persistent organic pollutant and by-product in the production of various chlorinated hydrocarbons that was previously used as a pesticide and component of transformer, hydraulic and heat-transfer liquids. Renal toxicity and carcinogenicity of HCBD observed in experimental animals (Kociba et al., 1977a; Kociba et al., 1977b; NTP, 1991) has been linked to GSH-mediated biotransformation in the liver, yielding 1-(glutathion-S-yl)-1,2,3,4,4-pentachlorobutadiene (PCBG), subsequent translocation of the GSH-conjugate to the kidneys and processing by GGT and dipeptidases to the corresponding cysteine S-conjugate, S-(1,2,3,4,4-pentachloro-1:3-butadienyl)-l-cysteine (PCBC) (Dekant et al., 1990). Uptake of PCBC into proximal tubule cells and renal cysteine-conjugate β-lyase mediated cleavage gives rise to reactive intermediates that may bind to tissue nucleophiles (Dekant et al., 1990).

Trichloroethylene (TRI) and tetrachloroethylene (PER) are haloalkenes that are widely used as industrial solvents, e.g., for degreasing metals and dry-cleaning fabrics. TRI is nephrotoxic and carcinogenic based on experimental evidence in laboratory animals and epidemiological human data, while PER is nephrotoxic and considered likely to be a human carcinogen (NTP, 1986, 1988; NTP, 1990; IARC, 2014). Similar to HCBD, TRI and PER are bioactivated to reactive intermediates via glutathione-*S*-transferase-mediated GSH conjugation in liver to form *S*-(1,2-dichlorovinyl)-glutathione (DCVG) and *S*-(1,2,2-trichlorovinyl)-glutathione (TCVG), respectively. Following transport to the kidney and renal processing of the GSH conjugates to *S*-(1,2-dichlorovinyl)-l-cysteine (DCVC) and *S*-(1,2,2-trichlorovinyl)-l-cysteine (TCVC), respectively, DCVC and TCVC are taken up into proximal tubule cells and bioactivated to a reactive thioketene via cysteine-conjugate β-lyases.

Hydroquinone (HQ) is an intermediate used in the chemical industry that has been identified as a nephrotoxin and renal carcinogen in rodents (NTP, 1989). It is metabolized in the liver by cytochrome P450 enzymes to 1,4-benzoquinone (1,4-BQ), which reacts with GSH to form 2-(glutathion-S-yl)HQ. Further oxidation and conjugation reactions with GSH addition lead to formation of 2,3,5-tris-(glutathion-S-yl)HQ (TGHQ), a potent nephrotoxic metabolite of HQ. It is thought that site-specific toxicity of TGHQ to S3 proximal tubule cells is linked to uptake in form of its cysteine conjugate, intracellular redox cycling and protein adduction (Labenski et al., 2011).

The industrial chemical bromobenzene produces hepatotoxicity and nephrotoxicity. Bromobenzene is bioactivated via cytochrome P450 enzymes to bromophenol and subsequently to 2-bromohydroquinone (BHQ), which reacts with GSH to form three positional isomers of 2-bromo-(glutathione-S-yl)hydroquinone as well as a nephrotoxic bisglutathione conjugate, 2-bromo-bis(glutathione-S-yl)hydroquinone. The organ-specific toxicity of bromobenzene in the kidney has been linked to transport of the GSH-conjugates to the kidney and conversion of the corresponding cystein conjugates. Covalent binding of BHQ and quinol-thiother-derived covalent protein adducts have been found in kidney tubule cells (Schnellmann et al., 1989; Rodeheaver and Schnellmann, 1991) and/or kidneys of rats exposed to BHQ (Kleiner et al., 1998).

4-Aminophenol, used as a photographic developer, for dyeing textiles, hair and furs, and an intermediate in the manufacture of pharmaceuticals and azo dyes, was shown to cause site-specific toxicity to the proximal tubule epithelium (Green et al., 1969). 4-Aminophenol is metabolized in the liver via GSH-dependent pathways, giving rise to toxic GSH conjugates, including 4-amino-3-(glutathion-*S*-yl)phenol, 4-amino-2,5-bis(glutathion-*S*-yl)phenol, and 4-amino-2,3,5(or6)-tris(glutathion-*S*-yl)phenol) (Klos et al., 1992). Delivery of the GSH-conjugates to the kidneys, processing by GGT and dipeptidases, uptake into S3 proximal tubule cells as cysteine conjugates, and intrarenal oxidation to electrophilic quinone imines that covalently bind to tissue nucleophils are considered to be responsible for 4-aminophenol nephrotoxicity (Klos et al., 1992; Dekant, 2001).

### 4.2 The Adverse Outcome Pathway of Renal Protein Alkylation Leading to Kidney Toxicity

#### Molecular Initiating Event: Protein Alkylation

*In vitro* and *in vivo* studies using radiolabeled compounds or immunochemical approaches provide clear evidence for covalent binding of chemically reactive intermediates of chemical stressors of this AOP to kidney proteins (Tables 7A–E). Covalent binding to proteins of the proximal tubule is consistent with the formation of reactive metabolites of the chemical stressors for this AOP, and corresponds to the site of uptake and/or bioactivation. Inhibition of metabolic pathways that lead to reactive metabolite formation has been shown to block covalent binding to renal proteins, as exemplified by inhibition of covalent protein binding by aminooxyacetic acid, which blocks β-lyase mediated cleavage of *S*-(pentachlorbutadienyl)-cysteine and S-(1,2-dichlorovinyl)-cysteine (Hayden and Stevens, 1990; van de Water et al., 1995). There is also evidence from studies on bromobenzene that scavenging of reactive metabolites by GSH inhibits covalent protein binding and protects from mitochondrial toxicity (Lau and Monks, 1987; Schnellmann et al., 1989), further supporting adduction of renal proteins as an initiating event required for nephrotoxicity induced by these compounds. Analyses of the subcellular localization of covalently bound proteins provide evidence that both cytosolic and mitochondrial proteins are targeted by reactive metabolites (Hayden and Stevens, 1990; Birner et al., 1994), consistent with the presence of both cytosolic and mitochondrial cysteine-conjugate β-lyases.

**TABLE 7A:** Evidence from *in vitro* and *in vivo* studies on **trichloroethylene** (TRI) and its metabolites *S*-(1,2-dichlorovinyl)-glutathione (DCVG) and *S*-(1,2-dichlorovinyl)-cysteine (DCVC) supporting the key events and qualitative concordance of KEs within this AOP (n/a = no data available).

		Qualitative concordance	
MIE/KE	Short Name	*In vitro*	Animals
MIE	Alkylation Protein	• Time- and/or dose-dependent covalent binding of [^35^S]-DCVC in rabbit renal cortical slices (Wolfgang et al., 1990), human proximal tubule cells (Chen et al., 1990), rat proximal tubule cells (van de Water et al., 1995) and mitochondria isolated from rat kidney cortex (Hayden and Stevens, 1990). • Inhibition of covalent protein binding by aminooxyacetic acid, which blocks β-lyase mediated formation of reactive metabolite from DCVC (Hayden and Stevens, 1990; van de Water et al., 1995)	• Covalent binding of radiolabel to renal protein following oral treatment of F344 rats and B6C3F1 mice with [^14^C]-TRI and [^14^C]-DCVC (Eyre et al., 1995). • Adduct formation by TRI blocked by aminooxyacetic acid pretreatment (Eyre et al., 1995).
KE1	Dysfunction, Mitochondria	• Dose-related decrease in complex II (succinate ubiquinone reductase) activity in rat proximal tubule cells treated with DCVC and protection by the β-lyase inhibitor aminooxyacetic acid (van de Water et al., 1995) • Time-dependent decrease in ATP content and O_2_ consumption induced by DCVC in rabbit renal cortical slices, accompanied by morphological alterations of mitochondria (Wolfgang et al., 1990) • Inhibition of state-3 respiration by DCVC in mitochondria isolated from rat kidney cortex, with effects blocked by aminooxy-acetic acid, which inhibitits β-lyase mediated formation of reactive metabolite from DCVC (Hayden and Stevens, 1990) • Decrease in mitochondrial membrane potential and extensively swollen mitochondria prior to decrease in ATP content in porcine proximal tubule cells LLC-PK_1_ treated with DCVC (Chen et al., 2001) • Decrease in mitochondrial respiration in rat kidney cells and mitochondria isolated from rat kidney treated with DCVG and DCVC (Lash et al., 1995) • DCVC produced depressed O_2_ consumption rates (basal and/or nystatin-stimulated) in renal proximal tubule fragments isolated from male F344 rats (Tyson et al., 1990) • Inhbition of state-3 respiration by TRI, DCVG and DCVC in mitochondria isolated from rat and mouse renal cortical cells (Lash et al., 2001) • Depletion of cellular ATP, inhibition of respiration and los of mitochondrial membrane potentia in rat and human rpoximal tubule cells treated with DCVC (Xu et al., 2008)	• Impaired response towards 2,4-dinitrophenol-stimulated respiration and ATP hydrolysis in kidney mitochondria isoated 4 h after administration of DCVC to rats (Stonard and Parker, 1971)
KE2	Increase, Cytotoxicity	• Proximal tubule damage in S3 segment of rabbit renal cortical slices exposed to DCVC, accompanied by loss of brush border and enzyme leakage (GGT, ALP, LDH) (Wolfgang et al., 1990) • Time- and dose-related LDH release induced by TRI, DCVG or DCVC in isolated rat renal proximal tubules, rat renal cortical cells (Tyson et al., 1990; Lash et al., 2001) and primary human proximal tubule cells (Chen et al., 1990) (Cummings et al., 2000; Cummings and Lash, 2000) • Cytotoxicity of DCVG and DCVC in rat kidney cells (Lash et al., 1995; Xu et al., 2008)	• Renal lesions in the form of cytomegaly and karyomegaly of renal tubular epithelial cells and toxic nephropathy in 13-weeks and 2-years gavage study of TRI in differents strains of rats, predominantly involving the inner cortex and oter stripe of the outer medulla (NTP, 1990; Mally et al., 2006). • Proximal tubule necrosis and inflammation in kidneys of male Swiss-Webster mice injected with DCVC (Vaidya et al., 2003)
AO	Occurrence, Kidney Toxicity	-	• Renal dysfunction and acute renal failure evidenced by increased urine volume and glucose, and markedly increased BUN in male Swiss-Webster mice injected with DCVC with increasing severity related to dose (Vaidya et al., 2003)

**TABLE 7B:** Evidence from human, animal and *in vitro* studies on **perchloroethylene** (PER) and its and its glutathione- and cysteine-*S*-conjugates TCVG and TCVC supporting the key events and qualitative concordance of KEs within this AOP (n/a = no data available).

		Qualitative concordance		
MIE/KE	Short Name	*In vitro*	Animals	Humans
MIE	Alkylation, Protein	n/a	• Detection of dichloroacetylated protein in kidney cytosol and mitochondria after a single dose gavage of PER (1,000 mg/kg body weight by gavage) to male Wistar rats using an antibody raised against Nε-(dichloroacetyl)-l-lysine (Pahler et al., 1998) • Dose-dependent formation of Nε-(dichloroacetyl)-l-lysine predominantly in kidney mitochondria in male and female Wistar rats exposed to 400, 40, and 10 ppm PER for 6 h (Pahler et al., 1999) • Detection of modified mitochondrial and cytosolic proteins in renal fractions from rats treated with [^14^C]tetrachloroethene (200 mg/kg) or TCVC (40 μmol/kg, iv) using an immunochemical approach and liquid scintillation spectroscopy (Birner et al., 1994)	n/a
KE1	Dysfunction, Mitochondria	• Inhibition of state 3 respiration in isolated renal cortical mitochondria from rats and mice by PER and TCVG (Lash et al., 2002)	n/a	n/a
KE2	Increase, Cytotoxicity	• Time- and concentration-dependent increases in LDH release induced by PER and TCVG in renal cell suspensions (Lash et al., 2002)	• Tubular cell karyomegaly and nephrosis in subchronic and chronic rodent toxicity studies (NTP, 1986)	• Severe acute tubular necrosis in a patient after accidental ingestion of 75 g PER (Choi et al., 2003)
AO	Occurrence, Kidney Toxicity	-	n/a	• Oliguric acute renal failure in a patient after accidental ingestion of 75 g PER (Choi et al., 2003) • End-stage renal disease in dry cleaning workers exposed to PER (Calvert et al., 2011)

**TABLE 7C:** Evidence from human, animal and *in vitro* studies on **hexachloro-1,3-butadiene (HCBD)** and its glutathione- and cysteine-*S*-conjugates (S-(pentachlorbutadienyl)glutathione (PCBG) and *S*-(pentachlorbutadienyl)-cysteine (PCBC)) supporting the key events and qualitative concordance of KEs within this AOP.

		Qualitative concordance	
MIE/KE	Short Name	*In vitro*	Animals
MIE	Alkylation, Protein	• Covalent binding of [^35^S]-PCBC to protein in mitochondria isolated from rat or rabbit kidney cortex (Hayden and Stevens, 1990; Groves et al., 1991; Brown et al., 1996), and inhibition of covalent protein binding by aminooxy-acetic acid, which blocks β-lyase mediated formation of reactive metabolite from PCBC (Hayden and Stevens, 1990).	• Covalent binding of [^14^C]-HCBD or metabolites to renal proteins of male and female rats administered and oral dose of 200 mg/kg bw of [^14^C]-HCBD (Birner et al., 1995).
				
KE1	Dysfunction, Mitochondria	• Inhbition of cellular respiration and depletion of ATP by PCBG in isolated rat renal epithelial cells associated with loss of cellular thiols. (Jones et al., 1986) • Concentration dependent disruption of mitochondrial membrane potential, inhibition of state 3 respiration, depletion of mitochondrial GSH and reduced ability to retain calcium in isolate rat renal cortical mitochondria exposed to PCBC (Wallin et al., 1987) • Inhbition of state-3 respiration by PCBC in mitochondria isolated from rat kidney cortex, with effects blocked by aminooxy-acetic acid, which inhibits β-lyase mediated formation of reactive metabolite from PCBC (Hayden and Stevens, 1990) • Concentration-dependent disruption of mitochondrial membrane potential and oxidation of pyrimidine nucleotides in isolated rat kidney mitochondria by PCBC (Brown et al., 1996) • Initial increase in mitochondrial respiration vial uncoupling of oxidative phosphorylation, followed by inhibition of state 3 respiration, cytochrome c-cytochrome oxidase and electron transport in rabbit renal proximal tubules exposed to PCBC (Schnellmann et al., 1987b) • Uncoupling of oxidative phosphorylation, followed by reduction of state 3 respiration and decrease in cellular ATP levels prior to cell death in isoated rabbit renal proximal tubules exposed to PCBC (Groves et al., 1991)	• Mitochondrial swelling in proximal tubule cells of rats treated with HCBD at 200 mg/kg bw (Ishmael et al., 1982)
KE2	Increase, Cytotoxicity	• Cytotoxicity of PDBG in isolated rat renal epithelial cells (Jones et al., 1986) • Concentration-dependent nephrotoxicity of PCBG in isolated perfused rat kidney evidenced by increases in urinary alkaline phosphatase and GGT and impaired glucose reabsoption (Schrenk et al., 1988) • Concentration-dependent toxicity of PCBC in isolated rabbit renal tubules (Jaffe et al., 1983)	• Renal tubular cell necrosis and/or regeneration in B6C3F1 mice receiving HCBC via diet for 2 or 13 weeks (NTP, 1991) • Renal tubule necrosis involving the pars recta of the proximal tubule in rats administered a single dose of [^14^C]-HCBD (200 mg/kg, per gavage) (Birner et al., 1995) • Proximal tubule necrosis in rats given a single i.p. dose of HCBD at 200 mg/kg bw (Ishmael et al., 1982) • Proximal renal tubule cell degeneration in rats in response to a single ip dose of HCBD, associated with increased urinary biomarkers indicative of kidney injury (Maguire et al., 2013) • Dose-dependent damage to the proximal tubules of the pars recta of Swiss-Webster male mice treated with PCBC (Jaffe et al., 1983)
				
AO	Occurrence, Kidney Toxicity	-	• Impaired renal function evidenced by increased sCrea and increased urinary glucose in rats in response to a single i.p. dose of HCBD (Maguire et al., 2013) • Impaired kidney function evidenced by decreased urine osmolality and reduced glomerular filtration rate in rats after a single i.p. dose of HCBD (Davis et al., 1980) • Increased plasma urea after dosing of rats with HCBD (200 mg/kg bw; i.p.) (Ishmael et al., 1982)

**TABLE 7D:** Evidence from animal and *in vitro* studies on **bromobenzene** and its metabolites 2-bromophenol and 2-bromohydroquinone (BHQ) supporting the key events and qualitative concordance of KEs within this AOP. (Data on human toxicity of bromobenzene are not available (EPA, 2009).).

		Qualitative concordance	
MIE/KE	Short Name	*In vitro*	Animals
MIE	Alkylation, Protein	• Dose- and time-dependent increase in covalently bound BHQ-equivalents to tubular protein in isolated rabbit proximal tubule cells exposed to [^14^C]-BHQ (Schnellmann et al., 1989; Rodeheaver and Schnellmann, 1991). • Inhibition of covalent binding by GSH protects from BHQ mediated inhibition of mitochondrial function (Schnellmann et al., 1989) • Covalent binding of [^14^C]-BHQ] equivalents to protein in homogenates from rat renal papillae, and inhibition by GSH (Lau and Monks, 1987)	• Covalent binding of [^14^C]-bromobenzene to kidney proteins in rats and mice (Reid, 1973) • Covalent binding of [^14^C]-bromophenol to renal proteins of male SD rats (Lau et al., 1984b) • Covalent binding of BHQ-equivalents to renal cortical protein in male SD rats administered a single dose of [^14^C]-BHQ; covalent binding and toxicity blocked by the GGT inhibitor acivicin (Lau and Monks, 1990). • Detection of quinol-thiother-derived covalent protein adducts in renal subcellular fractions of rats treated with BHQ (Kleiner et al., 1998)
				
KE1	Dysfunction, Mitochondria	• Decrease in respiration (nystatin-stimulated quabain sensitive oxygen consumption) and intracellular ATP content in rabbit proximal tubule cells exposed to BHQ linked to inhibition of state 3 respiration and subsequently inhibition of electron transport through cytochrome *c*-cytochrome oxidase (Schnellmann et al., 1987a; Schnellmann, 1989). • Induction of Ca^2+^ release from isolated pig renal cortical mitochondria by 2-Bromo-3-(*N*-acetylcystein-S-yl)hydroquinone (Vamvakas et al., 1992)	• Impaired mitochondrial function in kidney of Wistar rats treated with a single oral dose of bromobenzene (10 mmol/kg) as evidenced by decreased activities of tricarboxylic acid cycle enzymes (isocitrate dehydrogenase, α-ketoglutarate dehydrogenase, succinate dehydrogenase, malate dehydrogenase) and respiratory enzymes (NADH dehydrogenase and cytochrome c oxidase) (Vedi et al., 2014).
				
KE2	Increase, Cytotoxicity	• Time-dependent LDH release in isolated rabbit proximal tubule cells exposed to bromoquinone (Rodeheaver and Schnellmann, 1991) • Cytotoxicity of 2-bromo-3-(N-acetylcystein-S-yl)-hydroquinone in isolated rat renal cortex cells (Vamvakas et al., 1992)	• Necrosis of the proximal convoluted tubule induced by [^14^C]-bromobenzene in rats and mice (Reid, 1973) • Renal necrosis associated with increased BUN in male SD rats treated with 2-bromophenol with increasing severity related to dose (Lau et al., 1984b) • Severe proximal tubular necrosis in kidneys of male SD rats treated with BHQ (Lau et al., 1984a) • Significantly increased incidences of kidney lesions involving the proximal convoluted tubule in 90 days oral and inhalation studies on bromobenzene in rats and mice (summarized in (EPA, 2009))
				
AO	Occurrence, Kidney Toxicity	–	• Dose-related increase in BUN in mice treated with bromobenzene (Rush et al., 1984) • Dose-related increase in BUN in male SD rats treated with 2-bromophenol with increasing severity related to dose (Lau et al., 1984b) • Dose-related increase in BUN in male SD rats treated with BHQ with increasing severity related to dose (Lau et al., 1984a) • Impaired renal function induced by single dose of 2-bromophenol in female rats evidenced by increased BUN and reduced creatinine clearance (Bruchajzer et al., 2002) • Increase in BUN in rats treated with BHQ and BHQ derived GSH conjugates; nephrotoxicity prevented by the GGT inhibitor acivicin (Monks et al., 1985)

**TABLE 7E:** Evidence from animal and *in vitro* studies on **4-aminophenol (PAP)** and its nephrotoxic GSH conjugates supporting the key events and qualitative concordance of KEs within this AOP. Human toxicity data on PAP are not available (EPA, 2005). (n/a = no data available).

		Qualitative concordance	
MIE/KE	Short Name	*In vitro*	Animals
MIE	Alkylation, Protein	n/a	• Covalent binding of [^3^H]-PAP to kidney protein in male SD rats (Crowe et al., 1979) • Dose-related covalent binding of [^3^H]-PAP to kidney protein in male F344 rats (Fowler et al., 1993)
			
KE1	Dysfunction, Mitochondria	• Decreased respiration (nystatin-stimulated quabain sensitive oxygen consumption) and intracellular ATP content in isolated rabbit proximal tubules treated with PAP (Lock et al., 1993) • Inhibition of mitochondrial respiration (reduced O_2_ consumption rates) by PAP in proximal tubule fragments isolated from male F344 rats (Tyson et al., 1990) • Time- and dose-related inhibition of mitochondrial respiration (reduced O_2_ consumption rates) and decrease in intracellular ATP content induced by PAP in proximal tubule cells isolated from female SD rats (Li et al., 2005)	• Decreased activity of cytochrom b5 and NADPH cytochrom c reductase (complex III) indicative of impaired mitochondrial respiration in kidney of male SD rats treated with PAP (Crowe et al., 1979) • Significant decrease in O_2_ consumption in renal slices of PAP treated SD rats as compared to controls (Shao and Tarloff, 1996)
			
KE2	Increase, Cytotoxicity	• Time- and dose-related LDH release induced by PAP in isolated rabbit renal proximal tubules (Lock et al., 1993) (Tyson et al., 1990) • Dose- and time-dependent loss of cell viability in isolated rat kidney cortical cells induced by 4-amino-3-(glutathion-S-yl)phenol, 4-amino-2,5-bis(glutathion-S-yl)phenol, and 4-amino-2,3,5(or 6)-tris(glutathion-S-yl)phenol (Klos et al., 1992)	• Proximal tubule necrosis involving the pars recta in male F344 and female SD rats treated with a single dose of PAP or 4-amino-3-*S*-glutathionylphenol (Gartland et al., 1989; Fowler et al., 1991; Fowler et al., 1993; Shao and Tarloff, 1996)
			
AO	Occurrence, Kidney Toxicity	-	• Dose-related increase in BUN in male F344 and female SD rats treated with a single dose of PAP and 4-amino-3-*S*-glutathionylphenol (Fowler et al., 1991; Fowler et al., 1993) (Newton et al., 1982; Gartland et al., 1989; Shao and Tarloff, 1996)

Studies on tetrafluoroethene and its metabolite S-(1,1,2,2-tetrafluoroethyl)-l-cysteine (TFEC) in rats demonstrated acylation of renal proteins (Harris et al., 1992), with a high specificity for covalent binding to mitochondrial proteins (Hayden et al., 1991). Purification and NH_2_-terminal sequence analysis identified mitochondrial HSP60/P1-protein and HSP70-like protein (Mortalin) as major targets of TFEC (Bruschi et al., 1993), although it is still unclear if adduction of these proteins contributes to toxicity. As some compounds, such as the nontoxic acetaminophen analogue 3-hydroxyacetanilide, cause covalent binding in the absence of toxicity, it is now recognized that the binding pattern to certain cellular targets rather than the absolute level of binding may encode the biological response (Myers et al., 1995). Considering that different electrophiles preferentially attack different amino acid residues, it is also clear that different reactive metabolites will give rise to differential patterns of target protein modifications and cellular responses. For instance, bromobenzene and its hydroquinone metabolites appear to preferentially alkylate cysteine residues (Slaughter and Hanzlik, 1991), whereas the thioketenes formed by bioactivation of TRI and PER target both cysteine and lysine residues. Overall, specific proteins critical for subsequent cellular responses leading to proximal tubule toxicity remain to be identified. Moreover, the expected differential alkylation of proteins by various electrophiles highlights the necessity to further refine the AOP of renal protein alkylation leading to kidney toxicity based on future understanding of the contribution of different target proteins to toxic outcome.

#### Key Event 1: Mitochondrial Dysfunction

Evidence for mitochondrial dysfunction as a key event in this AOP comes primarily from a wide range of *in vitro* studies, consistently demonstrating inhibition of cellular respiration, depletion of ATP and disruption of mitochondrial membrane potential by chemical stressors of this AOP or their nephrotoxic metabolites (Tables 7A–E). These *in vitro* findings are supported by data from a limited number of *in vivo* studies, reporting mitochondrial swelling and/or decreased O_2_ consumption, decreased activity of tricarboxylic acid cycle enzymes and respiratory enzymes indicative of impaired mitochondrial respiration in kidneys of rats treated with a chemical stressor for this AOP (Crowe et al., 1979; Ishmael et al., 1982; Shao and Tarloff, 1996; Vedi et al., 2014). Importantly, the β-lyase inhibitor aminooxyacetic acid, which prevents β-lyase mediated cleavage of cysteine-*S*-conjugates to reactive intermediates, was shown to block both covalent binding and mitochondrial effects of *S*-(pentachlorbutadienyl)-cysteine and *S*-(1,2-dichlorovinyl)-cysteine (Hayden and Stevens, 1990; van de Water et al., 1995), supporting a causal link between reactive metabolite formation, protein adduction and mitochondrial toxicity.

#### Key Event 2: Increase, Cytotoxicity (Renal Tubular Cell)

It is generally accepted that interference with mitochondrial energy production may lead to cell death via apoptosis or necrosis. Mitochondrial toxicity is recognized as a critical event in drug-induced kidney injury induced by a wide range of chemicals (Gai et al., 2020). Proximal tubule cells highly depend on mitochondria to ensure an adequate ATP supply for active transporters expressed on the basolateral and brush border membrane of proximal tubular cells to facilitate tubular secretion and reabsorption. Inhibition of the triarboxylic acid cycle and the electron transport chain ultimately results in ATP depletion. In addition, opening of the mitochondrial permeability transition pore leads to mitochondrial dysfunction via mitochondrial depolarization, ATP depletion, release of Ca^2+^ from mitochondria, and inhibition of respiration–mitochondrial changes typically observed in response to chemical stressors of this AOP. There is also ample evidence from *in vitro* and *in vivo* studies in rodents for proximal tubule cell toxicity induced by the chemical stressors for this AOP and their respective nephrotoxic metabolites (Tables 7A–E).

#### Adverse Outcome: Kidney Toxicity

The link between proximal tubule injury and impaired kidney function has already been described in Section 2.3. Renal dysfunction and renal failure evidenced by increased urine volume, BUN or sCrea have been reported in experimental animals and humans exposed to chemical stressors of this AOP (Tables 7A–E).

### 4.3 Assessment of the Adverse Outcome Pathway of Renal Protein Alkylation Leading to Kidney Toxicity

#### Biological Plausibility

The covalent binding hypothesis of chemical toxicity, which goes back to the early 1970s, is a well-established principle in toxicology. The sequence of events leading from bioactivation of a xenobiotic to a reactive electrophile, which covalently binds to proteins and alters protein function, to toxicity and cell death is experimentally well supported. There is ample evidence that covalent binding to renal proteins is causally linked to the nephrotoxicity of a range of chemicals. Although there is yet a paucity of information on specific target proteins and their link to impaired mitochondrial function and cell death, the level of confidence in the biological plausibility of key event relationships (KERs) within the proposed AOP can be considered as high (Table 8).

**TABLE 8:** Weight-of-evidence analysis of KERs in the adverse outcome pathway of renal protein alkylation leading to kidney toxicity.

KE Upstream	KE Downstream	Weight of evidence (WoE) for KER			
		Biological Plausibility	Essentiality	Empirical support	Overall WoE
Alkylation, Proteins	Dysfunction, Mitochondria	moderate	high	moderate	high
Dysfunction, Mitochondria	Increase, Cytotoxicity	high	high	high	high
Increase, Cytotoxicity	Occurrence, Kidney Toxicity	high	high	high	high

#### Essentiality of Key Events

Essentiality of the MIE in this AOP is supported by a number of *in vitro* and *in vivo* studies, which demonstrate that inhibition of covalent binding of reactive metabolites to cellular proteins (via enzyme inhibitors that block reactive metabolite formation or scavenging of electrophiles by GSH) protects from mitochondrial toxicity and/or toxicity of chemical stressors for this AOP (Monks et al., 1985; Schnellmann et al., 1989; Hayden and Stevens, 1990; Lau and Monks, 1990; van de Water et al., 1995). As the entire sequence of KE and the AO can be blocked by inhbition of the MIE, there is clear evidence that covalent binding to proteins is essential (Table 8). Using inhibitors of specific mitochondrial processes, Xu et al. (2008) showed that mitochondrial dysfunction is an essential step in cell injury induced by DCVC in human proximal tubule cells (Xu et al., 2008). While these experimental data support the biologically plausible link between adduction of mitochondrial proteins, mitochondrial dysfunction and toxicity, it is important to recognize that covalent protein binding may well affect other organelles and thereby cause toxicity by mechanisms other than mitochondrial dysfunction, e.g. through induction of ER stress. This, however, does not contest the causal relationship between covalent protein binding, mitochondrial dysfunction and toxicity and thus the validity of mitochondrial dysfunction as a KE, but rather suggests that several KEs may branch out of the MIE (covalent protein binding) and combine to cause nephrotoxicity as the AO. The relative contribution of such branches to the overall outcome may differ between chemical stressors and depend on several factors, including the chemical reactivity of the stressor or its metabolite, the dose over time and consequently the target proteins affected by adduction, and their essentiality for cell homeostasis. These considerations are vital when it comes to application of AOPs for toxicity prediction, as the quantitative relationships between the measurable and essential, but yet mechanistically poorly defined MIE and downstream KEs including the AO may not be universal for all stressors that trigger the MIE, as exemplified by the poor correlation between covalent protein binding and hepatotoxicity of the acetaminophen analogue 3-hydroxyacetanilide.

#### Empirical Evidence: Dose-Response and Temporal Concordance

There is ample experimental evidence to support the temporal sequence of events in this AOP. In kidneys of rodents treated with a single i.p. dose of bromobenzene, covalent protein binding preceded the onset of histopathological lesions (Reid, 1973). Similarly, in isolated rabbit proximal tubules treated with bromohydroquinone, covalent binding to tubular protein and mitochondrial changes occurred rapidly within 15 min and preceded loss of cell viability (Schnellmann et al., 1987a; Schnellmann et al., 1989). Inhibition of mitochondrial respiration and loss of ATP was also shown to occur prior to cell death in rabbit proximal tubule cells treated with 4-aminophenol (Lock et al., 1993).

In a 12 h time-course study of DCVC toxicity in rabbit renal cortical slices, a time-dependent increase in covalent binding of [^35^S]DCVC was observed between 5 and 120 min. These effects were followed by a decline in mitochondrial function, oxygen consumption and ATP content, which manifested at 4–8h, and histological evidence of S3 proximal tubule injury after 8 h exposure (Wolfgang et al., 1990). Moreover, pulsed versus continuous exposure to DCVC demonstrated that 30 min exposure to DCVC, in which substantial covalent binding occured, was sufficient to trigger proximal tubule toxicity (Wolfgang et al., 1990). In a similar study on the time-course of DCVC toxicity in porcine proximal tubule cells, a decrease in mitochondrial membrane potential was evident at 4h, whereas biochemical changes associated with apoptosis (cytochrome C release, caspase-3 activity, DNA fragmentation) and decreases in cellular ATP manifested at 6–8 h (Chen et al., 2001). Support for mitochondrial dysfunction as an early event that precedes proximal tubule injury also comes from studies on the hexachorobutadiene metabolite *S*-pentachloro-1,3-butadienyl)-l-cysteine (PCBC) in rabbit renal proximal tubules. Here, mitochondrial changes were recorded within 15 min of exposure, while a decrease in cell viability was evident after 60 min of exposure to PDBC (Schnellmann et al., 1987b).

Although no detailed *in vivo* dose-response studies are available, covalent binding and proximal tubule damage, or mitochondrial changes, proximal tubule cell necrosis and impaired function were all observed in rats following a single oral or i.p. dose of HCBD at 200 mg/kg bw (Ishmael et al., 1982; Birner et al., 1995). Similarly, covalent binding of [^14^C]-bromobenzene or [^14^C]-2-bromophenol to mouse or rat kidney proteins *in vivo* was recorded at the same dose that resulted in histological evidence of kidney injury (Reid, 1973) or impaired kidney function (Lau et al., 1984a). *In vitro* studies on DCVC indicate a high concordance between concentrations that cause covalent protein binding, mitochondrial effects and cytotoxicity (van de Water et al., 1995).

Collectively, the available *in vitro* and *in vivo* studies conducted using chemical stressors for this AOP support the temporal sequence of KE and demonstrate effects on KEs across the entire AOP at equal doses/concentrations of the stressor. Based on the criteria for assessing AOP (Box 1), the level of confidence for temporal and dose-response concordance can thus be regarded as high (Table 8).

#### Weight-Of-Evidence Analysis

Based on biological plausibility and empirical support, the overall weight-of-evidence of KERs in this AOP can be considered as high (Table 8).

### 4.5 Quantitative and Temporal Understanding of Key Event Relationships

Based on the available literature, there is at present little or no quantitative information on the response-response relationship between two pairs of KEs in this AOP.

#### KER1: Protein Alkylation Leading to Mitochondrial Dysfunction

There is as yet no information regarding the quantitiative response-response relationship between covalent protein binding and mitochondrial dysfunction or toxicity in general. This stems from an insufficient mechanistic understanding of which specific target proteins are critical for toxicity. It is widely appreciated that total covalent protein binding cannot be utilized as a good predictor of cytotoxic potential, but rather that selective binding to critical cellular targets may drive the outcome (Cohen et al., 1997 *).*

#### KER2: Mitochondrial Dysfunction Leading to Proximal Tubule Cytotoxicity

As outlined in Section 2.5, it is evident that proximal tubule cells depend on cellular respiration and mitochondrial ATP production to provide energy for active transport of solutes. There is, however, no systematic assessment as to how much decline in mitochondrial function or ATP depletion and for how long may be tolerated by a proximal tubule cell before it commits to apoptosis or necrosis.

#### KER3: Proximal Tubule Cytotoxicity Leading to Kidney Toxicity

Proximal tubule cytotoxicity is a common KE across all three AOPs discussed here. The link between proximal tubule toxicity and impaired kidney function is well established, although it is less clear how much cell killing over time is needed to cause functional impairment. For further considerations, the reader is referred to Sections 2.5 and 3.5.

## 5 Towards a Network of Adverse Outcome Pathways for Nephrotoxicity and Considerations for Implementation of Adverse Outcome Pathways for Safety Assessment

**FIGURE 8:**
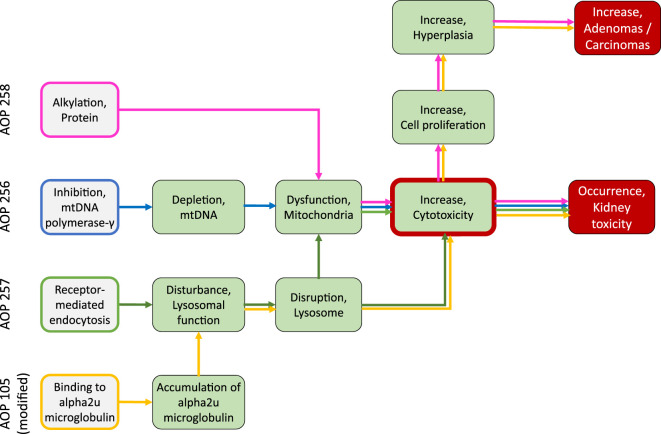
An initial network of AOPs leading to kidney toxicity, with mitochondrial dysfunction and renal tubular cell cytotoxicity as common KEs. Sustained renal tubular cell cytotoxicity is also recognized as an important trigger for regenerative cell proliferation, resulting in hyperplasia and ultimately renal tumors. While this has been proposed as an AOP in itself with cytotoxicity irrespective of the primary insult as the MIE, this AOP can be initiated by chemicals that bind to alpha_2u_ macroglobulin in serum in male rats (leading to accumulation of alpha_2u_ macroglobulin in lysosomes of renal tubular cells, associated with disturbance of lysosomal function and ultimately disruption of lysosomes) but also by chemicals that cause sustained cytotoxicity through protein alkylation. The AOP “Alpha_2u_-microglobulin cytotoxicity leading to renal tubular adenomas and carcinomas (in male rat)” developed by Charles Wood (US EPA) and published in AOPWiki (https://aopwiki.org/aops/105) was slightly extended to highlight overlapping KEs between alpha_2u_ nephropathy and the AOP of receptor-mediated endocytosis leading to kidney toxicity. The colored arrows indicate the pathways that lead from a specific MIE through shared KEs to the respective AO in kidney. While cytotoxicity is central KE in all presented AOPs, it is the extent of cytotoxicity over time that is expected to determine the AO. Note that - with the exemption of “Binding to alpha_2u_ macroglobulin” which occurs in serum–all MIEs and KEs shown relate to renal tubule cells or renal tubules.

Herein, we describe a set of AOPs for kidney injury that are triggered by different MIEs but involve proximal tubule toxicity as a common KE. Through shared KEs, our AOPs tie in with a previously established AOP “Alpha2u-microglobulin cytotoxicity leading to renal tubular adenomas and carcinomas (in male rat)” (https://aopwiki.org/aops/105) to build a first network of kidney related AOs (Figure 8), which is expected to be expanded progressively as further AOPs are being developed and validated. Eventually, a comprehensive AOP network may then provide a unique basis for the identification of a battery of *in vitro* and/or *in vivo* endpoints that cover the entire mechanistic landscape of chemically-induced kidney injury and can be integrated with endpoints relevant to other target organs into an integrated testing strategy to collectively address repeated dose toxicity. While the AOP on skin sensitisation (OECD, 2014) convincingly demonstrates how mechanistic information systematically captured in form of AOPs can be translated into new test guidelines for a specific hazard endpoint, it is evident that identification and characterization of potential health harzards that arise from repeated exposure and that may affect a broad range of targets of toxicity is increasingly more complex and is still in its infancy. The AOPs developed and evaluated here in view of identification of mechanistically relevant endpoints for renal safety assessment highlight a number of open issues that need to be discussed and addressed by the scientific community on the way to implementation of AOP based testing strategies for assessment of repeated dose toxicity, particularly for regulatory decision making beyond hazard identification.

The AOPs discussed here cover fairly well established mechanisms by which certain chemicals or drugs are thought to cause nephrotoxicity. Compared to other mechanisms or mode of actions that are less well defined, there is thus a wealth of data derived from multiple stressors to support the MIEs, KEs and KERs. While the overall confidence in these AOPs can be considered as high, there are still data gaps, as exemplified by the as yet insufficient mechanistic understanding of the causal link between alkylation of specific target proteins of reactive metabolites and mitochondrial dysfunction/toxicity. For AOPs to serve as a mechanistic framework to derive suitable endpoints for hazard identification, it may however not be necessary to fully understand and describe in detail all the molecular and cellular events and their causal relationships. Rather, a simple representation of the AOP by a few essential, generalized KEs using harmonized KE umbrella terms may present a pragmatic approach. Thus, even less well defined adverse outcome pathways may be integrated into an AOP network to ensure full coverage of the mechanistic landscape of an adverse outcome, which is essential for future testing approaches. Omitting pathways relevant to a particular health hazard because they are mechanistically poorly understood may otherwise hold the risk of creating critical gaps in future test strategies, which may leave some chemical hazards undetected. It thus appears equally important to integrate as yet ill-defined mechanisms with a low level of confidence as well as well-established mechanisms to obtain a wholistic network view of pathways leading to an adverse outcome. It may, however, be helpful to indicate the level of confidence or uncertainties in the graphical representations of AOPs, including the network view.

On the other hand, development of quantitative AOPs for toxicity prediction may require more specified KE terms rather than KE umbrella terms and also consider potential modulating events. Again, this is exemplified by the poor predictivity of total protein alkylation for toxicity. Understanding which specific protein targets contribute to perturbation of downstream events (in this case mitochondrial dysfunction, cytotoxicity, kidney injury), and to which extent, would be required to allow quantitative predictions. It is conceivable that the overall adverse effect initiated by protein alkylation may involve several pathways in parallel (e.g., covalent binding to ATP synthase leading to ATP depletion; covalent binding to protein thiols involved in redox regulation leading to impaired antioxidant defense). There should be consensus on whether such parallel pathways, that are likely to be affected by all chemical stressors but perhaps to a varying degree, should all form individual AOPs or rather an AOP family tree subsummized under a more generalized MIE term.

Similarly, there needs to be consensus on how to define and represent AOPs that operate in different target organs, such as protein alkylation, which may also cause hepato- and nephrotoxicity, or inhbition of mtPol γ, which has been linked to neurodegeneration, myopathy, cardiotoxicity and hepatotoxicity in addition to nephrotoxicity (Figure 3). Particularly when it comes to quantitative description of KERs, it needs to be considered that the tissue-specific response to a molecular initiating event may depend on the biological context of the cell and organ affected. For instance, the response to inhibition of mtDNA polymerase γ may depend on the rate of mitochondrial biogenesis, mtDNA content and energy demand on the cellular level, as well as on the functional reserve and regenerative capacity of the organ, all of which are tissues-specific (Figure 3). Despite a similar effect on mtPol γ, mitochondrial dysfunction and subsequent events are expected to have more detrimental effects in tissues with a high rate of mitochondrial biogenesis, a high energy demand, low functional reserve and capacity to regenerate. In addition, sex, age, and other susceptibility factors are likely to influence the quantitative KERs. Similarly, the temporal scale of effects may vary between tissues, and potential temporal delays between the MIE and the first KE in this AOP are important to consider when developing KE related endpoints for toxicity testing.

In our AOP-257 we designated receptor-mediated endocytosis as the MIE. As endocytosis facilitates uptake of stressors into the cell, it may rather be seen as part of the toxicokinetics of the stressors rather than an element of an AOP. However, as receptor-mediated endocytosis directs ligands to the lysosomal compartment, it is considered essential for down-stream lysosomal events to occur. However, this illustrates that strict dissociation of toxicokinetics and MIEs at the chemical-biological interface may not always be straightforward.

Finally, while AOPs are by definition chemically agnostic, it is important to realize that empirical support comes primarily from (presumed) chemical stressors of the AOP. Similarly, quantitative KERs are likely to be derived through use of chemical stressors, which poses a source of uncertainty as chemicals often act by more than one mechanism or pathway. An example is hydroquinone, which may covalenty bind to proteins but may also cause oxidative tress through redox cycling. As the relative contribution of multiple pathway to the overall outcome is rarely known even for toxicologically well-characterized chemicals, prediction of the toxicity of chemicals that act by more than one AOP will present a major scientific challenge.

## Data Availability

The original contributions presented in the study are included in the article/Supplementary Material, further inquiries can be directed to the corresponding author.

## Abbreviations

AOP, Adverse Outcome Pathway; AO, Adverse Outcome; BHQ, 2-Bromohydroquinone; BUN, Blood Urea Nitrogen; DCVC, S-(1,2-Dichlorovinyl)-cysteine; DCVG, S-(1,2-Dichlorovinyl)-glutathione; HCBD, Hexachloro-1,3-butadiene; KE, Key Event;KER, Key Event Relationship; MIE, Molecular Initiating Event;PAP, 4-Aminophenol; PCBC, S-(Pentachlorbutadienyl)-cysteine;PCBG, S-(Pentachlorbutadienyl)-glutathione; PER,Perchloroethylene; sCrea, Serum Creatinine; TDF, tenofovirDipivoxil Fumarate; TRI, trichloroethylene; TCVC, S-(1,2,2-Trichlorovinyl)-cysteine; TCVG, S-(1,2,2-Trichlorovinyl)- glutathione.
